# Graphene Sensor Arrays for Rapid and Accurate Detection of Pancreatic Cancer Exosomes in Patients’ Blood Plasma Samples

**DOI:** 10.1021/acsnano.3c01812

**Published:** 2023-07-20

**Authors:** Tianyi Yin, Lizhou Xu, Bruno Gil, Nabeel Merali, Maria S. Sokolikova, David C. A. Gaboriau, Daniel S. K. Liu, Ahmad Nizamuddin Muhammad Mustafa, Sarah Alodan, Michael Chen, Oihana Txoperena, María Arrastua, Juan Manuel Gomez, Nerea Ontoso, Marta Elicegui, Elias Torres, Danyang Li, Cecilia Mattevi, Adam E. Frampton, Long R. Jiao, Sami Ramadan, Norbert Klein

**Affiliations:** † Department of Materials, Imperial College London, London SW7 2AZ, U.K.; ‡ ZJU-Hangzhou Global Scientific and Technological Innovation Center, Zhejiang University, Hangzhou 311200, China; § Hamlyn Centre, Imperial College London, London SW7 2AZ, U.K.; ∥ Oncology Section, Surrey Cancer Research Institute, Department of Clinical and Experimental Medicine, FHMS, University of Surrey, The Leggett Building, Daphne Jackson Road, Guildford GU2 7WG, U.K.; ⊥ HPB Surgical Unit, Royal Surrey NHS Foundation Trust, Guildford, Surrey GU2 7XX, U.K.; # Minimal Access Therapy Training Unit (MATTU), University of Surrey, The Leggett Building, Daphne Jackson Road, Guildford GU2 7WG, U.K.; ¶ Facility for Imaging By Light Microscopy, Imperial College London, London SW7 2AZ, U.K.; ∇ Department of Surgery & Cancer, Imperial College London, Hammersmith Hospital Campus, London W12 0NN, U.K.; ⊗ HPB Surgical Unit, Imperial College Healthcare NHS Trust, Hammersmith Hospital, London W12 0HS, U.K.; ■ FTKEE, Universiti Teknikal Malaysia Melaka, 76100 Durian Tunggal, Melaka, Malaysia; ▲ Graphenea Semiconductor, Paseo Mikeletegi 83, San Sebastián ES 20009, Spain; ● Research Center, The Seventh Affiliated Hospital, Sun Yat-sen University, Shenzhen 518107, China

**Keywords:** graphene field-effect transistors, PDAC cancer, biosensor, GPC-1, exosomes

## Abstract

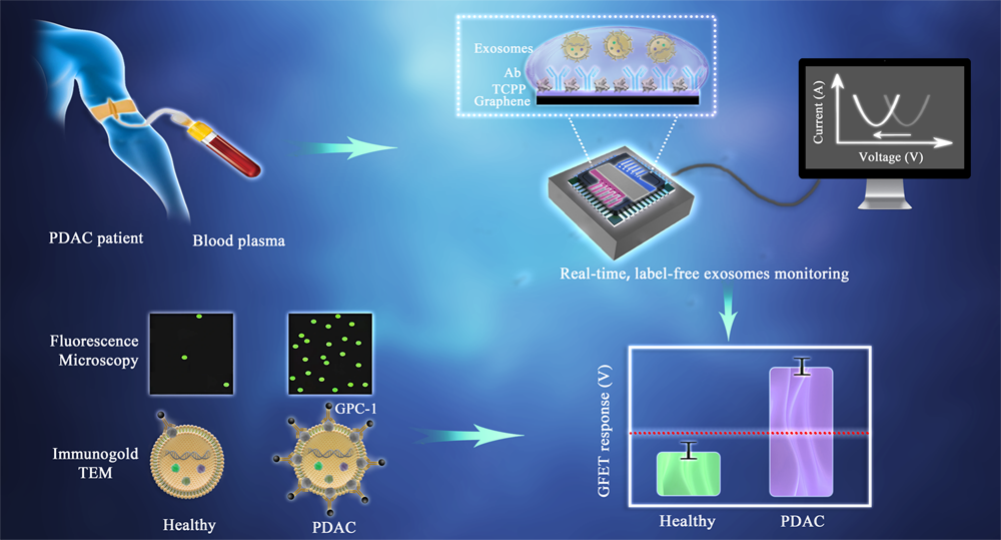

Biosensors based on graphene field effect transistors (GFETs) have the potential to enable the development of point-of-care diagnostic tools for early stage disease detection. However, issues with reproducibility and manufacturing yields of graphene sensors, but also with Debye screening and unwanted detection of nonspecific species, have prevented the wider clinical use of graphene technology. Here, we demonstrate that our wafer-scalable GFETs array platform enables meaningful clinical results. As a case study of high clinical relevance, we demonstrate an accurate and robust portable GFET array biosensor platform for the detection of pancreatic ductal adenocarcinoma (PDAC) in patients’ plasma through specific exosomes (GPC-1 expression) within 45 min. In order to facilitate reproducible detection in blood plasma, we optimized the analytical performance of GFET biosensors via the application of an internal control channel and the development of an optimized test protocol. Based on samples from 18 PDAC patients and 8 healthy controls, the GFET biosensor arrays could accurately discriminate between the two groups while being able to detect early cancer stages including stages 1 and 2. Furthermore, we confirmed the higher expression of GPC-1 and found that the concentration in PDAC plasma was on average more than 1 order of magnitude higher than in healthy samples. We found that these characteristics of GPC-1 cancerous exosomes are responsible for an increase in the number of target exosomes on the surface of graphene, leading to an improved signal response of the GFET biosensors. This GFET biosensor platform holds great promise for the development of an accurate tool for the rapid diagnosis of pancreatic cancer.

## Introduction

Pancreatic cancer (PC) is the second most deadly cancerous disease and the seventh-leading cause of cancer deaths worldwide, with 5-year survival rates estimated to be below 7.3% and approaching zero for patients with advanced pancreatic cancer.^1^ Despite the recent progress in cancer diagnosis and treatment, the overall 5-year survival rate in the case of PC has only marginally improved over the past decade.^2,3^ A critical factor in the poor outcomes is the lack of early symptoms. Most patients with symptoms present for medical evaluation only when the cancer is at an advanced stage. However, the likelihood of survival can be significantly improved if cancer is detected at an early stage. Therefore, early detection and diagnosis are crucial to improving outcomes.

Screening programs for PC can result in a decreased incidence and mortality. Imaging techniques such as computed tomography (CT) and magnetic resonance imaging/cholangiopancreatography (MRI/MRCP) are primary methods used for the detection and evaluation of pancreatic tumors in clinics. However, the use of such methods to screen the general population for PC is not practical or cost-effective due to its low incidence rate. Recently, liquid biopsies that use biomarkers such as circulating tumor DNA (ctDNA),^4−6^ circulating tumor cells (CTCs),^7^ exosomes,^8,9^ and microRNAs (miRNAs)^10,11^ presented in body fluids have emerged as promising approaches for early PC diagnostics. Among these biomarkers, exosomes are small extracellular vesicles (30–200 nm) released in high quantities by healthy and tumor cells via the endocytic pathway, which enables cell-to-cell communication and cargo transfer. There is increasing evidence that protein markers on exosomal pancreatic cancer-initiating cells (PCICs) are promising for the early detection of PC. Previous studies have reported that GPC-1 is specifically enriched on cancer-cell-derived exosomes.^12,13^ It has been found that PC patients express higher levels of GPC-1 on their exosomes than healthy controls with high specificity and sensitivity for PC.^14^ Other exosome-based proteins such as epidermal growth factor receptor (EGFR)^4^ and epithelial cellular adhesion molecule (EpCAM)^15^ also show high accuracy and selectivity for PC detection.

Current methods to detect specific exosomes, such as the Western blot, enzyme-linked immunosorbent assay (ELISA),^16^ and flow cytometry,^17^ usually take a long time to complete from sampling to results, and require complicated processing steps. In recent years, many sensor technologies have been developed to enhance the limit of detection (LOD) of assays for exosome detection, including potentiometric sensors,^18^ electrochemical methods,^19−21^ capacitive sensors,^22^ fluorescence methods,^23^ and surface-enhanced Raman scattering.^23,24^ There are only a few studies that specifically detect exosomal biomarkers from pancreatic patients’ samples.^24−26^ However, existing methods still lack the specificity and capability to detect an early stage of cancer. Therefore, a direct, accurate, and highly sensitive diagnosis platform with the capability for the specific detection of pancreatic cancer in clinical samples at an early stage is urgently needed.

Graphene field-effect transistors (GFETs) have emerged as a promising platform for the early diagnosis of diseases.^27−30^ A graphene monolayer consists of a carbon layer that is one atom thick that exhibits a strong response to charged molecules present on its surface. Furthermore, graphene is biologically compatible and can be directly functionalized without the need for new functionalization steps or damaging its sp2 network. Graphene growth via chemical vapor deposition allows the large-scale production of GFET sensors using standard CMOS-compatible wafer processes which enable a dramatic miniaturization of chip footprints with microwatts of power per sensor, without sacrificing performance in comparison to larger chips. Furthermore, GFET biosensors have been demonstrated to be capable of detecting a variety of biological species, including nucleic acids,^31−34^ small biomolecules such as glucose,^35^ dopamine,^36^ amino acids,^37^ proteins,^38^ exosomes,^39,40^ viruses,^41,42^ and other disease biomarkers. However, to our knowledge, no previous study has used the GFETs platform for the detection of pancreatic cancerous exosomes in clinical samples.

**Figure 1:**
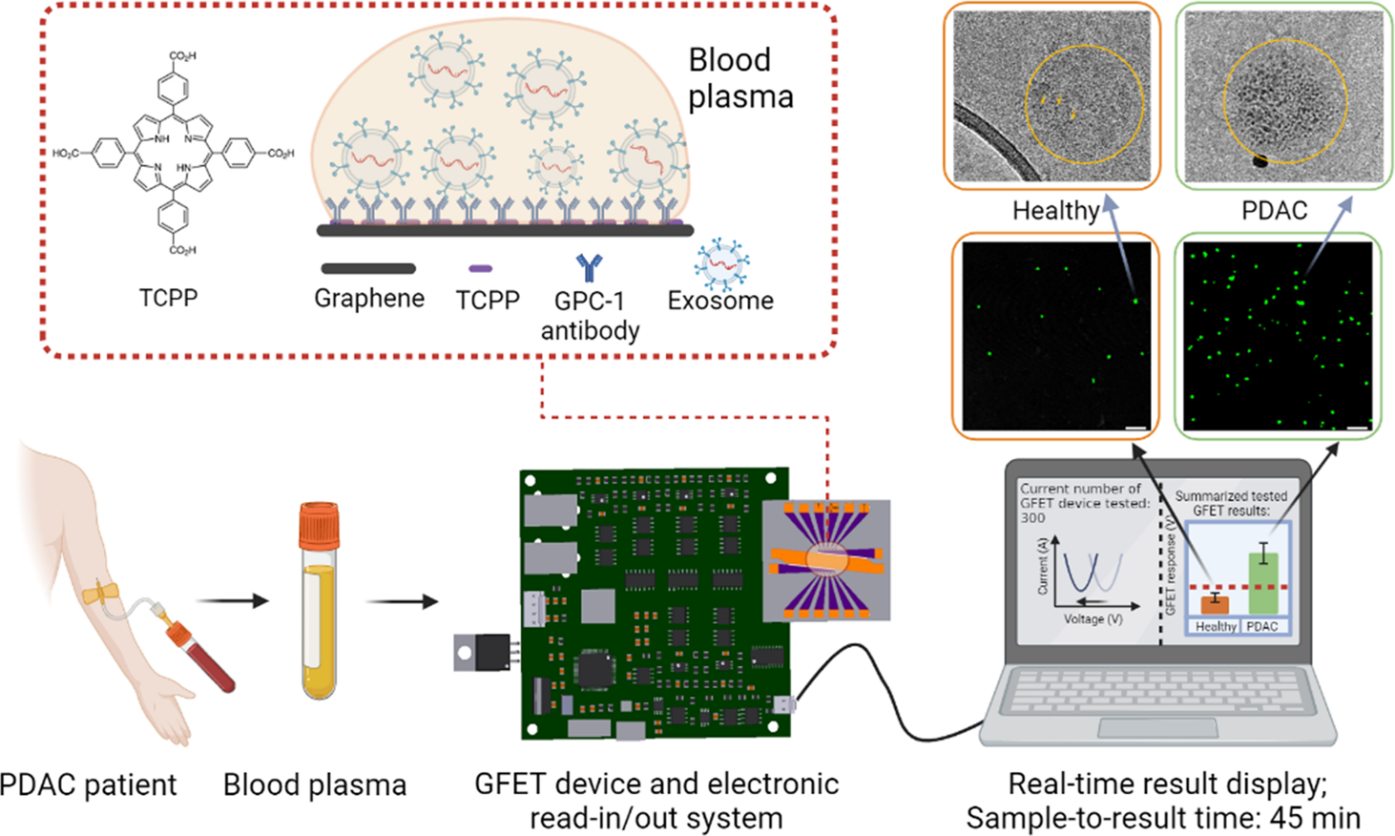
Schematics of detection of PDAC exosomes using GFETs with portable electronics and real-time detection results. The total detection time from applying blood plasma on the GFETs to results is less than 45 min. The zoomed in area shows schematics of the functionalization of graphene with TCPP^43^ and the GPC-1 antibody. The middle-right images are fluorescence images showing higher density of exosomes on the GFET surface for the PDAC patients’ samples than the healthy controls. The top-right images are TEM images with immunogold labeling with GPC-1 to compare the GPC-1 protein expressions on healthy and cancerous exosomes.

Here, we developed an on-chip-based POCT (point-of-care testing) GFET sensor platform for the detection of pancreatic cancerous exosomes in patient plasma (Figure 1). The platform consists of GFET sensor arrays with liquid gate electrodes integrated on the chip. A portable read-in/out electronic system was built to measure the real-time electrical response from the GFET sensors for 12 channels on one chip simultaneously (Section S1). We used this platform to detect GPC-1 in plasma from 26 patients using a 20 μL drop within 45 min. Our GFET technology is clearly shown to be able to discriminate between samples from healthy controls and PDAC patients. We observed a significant increase in the cancerous exosome binding to the sensor surface compared with healthy exosomes, even when they were both at the same volume concentration level in the plasma. We compared the GFET results to MRI and CT data to evaluate the performance of the proposed GFET sensor for clinical testing. Furthermore, this platform is portable and can be easily integrated to simultaneously detect multiple cancerous biomarkers in real time. Thus, electrical detection using GFETs could be a promising diagnosis platform for the early diagnosis of pancreatic cancer and other diseases.

## Results and Discussion

### Design and Characterization of the GFET Sensor Array for Clinical Testing

Although it has been demonstrated that single GFETs are able to detect biomarkers at femtomolar concentration under laboratory conditions,^32−34^ the level of consistency of the GFET response for a larger number of sensors under the condition of a clinical study has barely been reported yet. Biodetections with GFET sensors are often limited to measurements in nonphysiological solutions with low ionic strength due to the Debye screening effect. To realize the detection in blood plasma, we optimized the analytical performance of our GFET biosensors for pancreatic cancer exosome detection via the application of an internal control channel and the development of an optimum test protocol. Furthermore, since overcoming the issues of reproducibility of GFETs fabrication and the uniformity of electrical response from device-to-device and chip-to-chip are important in clinical testing, we followed strict quality control methods in graphene production. In addition, we set up an exclusion criteria before clinical testing to improve consistency and the reliability of detection. Moreover, the advantage of graphene arrays was emphasized for the reliable detection of ions,^44^ aiming to overcome the shortcoming of the graphene technology. Here we demonstrate the necessity of GFET arrays and negative control subarrays for the meaningful detection of exosome biomarkers in clinical samples from cancer patients.

**Figure 2:**
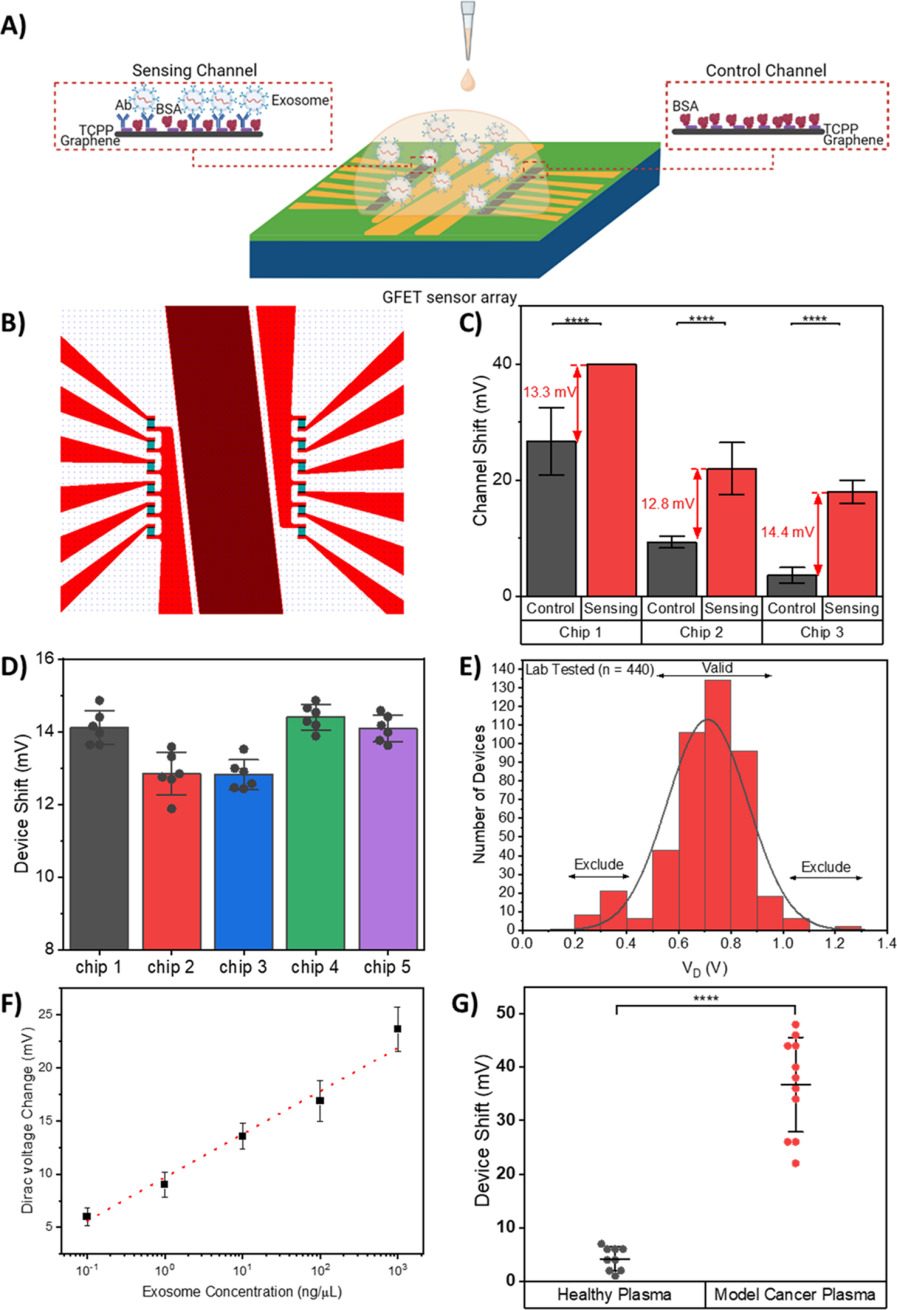
Design, characterization, and performance assessment of the on-chip integrated GFET device. (A) Schematics of the on-chip integrated GFET biosensor platform with the antibody functionalized channel as the sensing channel and a channel without antibody functionalization as the control channel. (B) Illustration of the layout of on-chip integrated GFET sensor, designed with 6 devices each on the left and right sides. These devices are separated by the central common-on-chip integrated Au electrode. (C) GFET device shift of the control and sensing channels on three GFET chips tested for the same concentration of exosomes in buffer, illustrating the importance of control channel. The GFET device detection signal is calculated based on the difference between the GFET response from control and sensing channels. Error bars are determined by the standard deviation of multiple device measurements, 5 ≤ *n* ≤ 6 for each reading. (D) GFET device detection signal comparison between the five chips tested for the same concentration of exosomes in buffer, illustrating low device-to-device variation and high chip-to-chip reproducibility of the GFET biosensor. Error bars are determined by the standard deviation of multiple device measurements, 4 ≤ *n* ≤ 6 for each reading. (E) Device exclusion criteria for GFET reproducibility and validity. Histogram of Dirac voltage for lab-tested GFET devices with Gaussian distribution curve. Device exclusion criteria were set based on 444 lab-tested GFET devices, where the Dirac voltage for lab-tested devices was *V*_D_ = 0.71 ± 0.24 V. (F) Calibration curve of the GFET biosensor for detection of various concentrations of model cancerous exosomes in buffer. Error bars are determined by the standard deviation of multiple device measurements, 4 ≤ *n* ≤ 24 for each reading. (G) Measurement results for the GFET biosensor with healthy plasma and model cancer plasma, illustrating excellent selectivity of the GFET biosensor. (****P < 0.0001). Data are mean ± standard deviation (s.d.).

Our GFET biosensor is an on-chip integrated sensor array comprising 12 individual GFET devices. These devices are separated into two sections by a central common-on-chip integrated Au electrode. The on-chip integrated liquid gate allows the simultaneous measurement of all 12 GFET devices for the detection of the same sample solution (Figure 2B and Figure S1). Due to the complex physiological environment of blood plasma with high concentration of other nondesired species, the direct detection with blood plasma is very challenging. The noise produced by nonspecific binding interactions will lead to a reduction in selectivity and limiting detection sensitivity. Therefore, we used two separate sections of the devices that can be classified as a sensing channel (6 GFETs devices in parallel on the left-hand side) and a control channel (6 GFETs devices in parallel on the right-hand side). The sensing channels are functionalized with GPC-1 antibodies for specific target detection in sample solution, whereas the control channels lack any antibody functionalization aiming for the detection of background signals in the same sample solution (Figure 2A). The use of control and sensing channels can help in distinguishing between signals arising from specific target binding and interfering signals caused by background buffer or unwanted nonspecific binding events on the graphene surface. Hence, the accuracy and reliability of the detection of specific targets can be improved.

To illustrate the role of internal control in improving the accuracy of the detection, we spiked 10 ng/μL exosomes in the buffer and tested the GFETs device response from multiple chips. As shown in Figure 2C, the introduction of exosomes results in large and inconsistent shifts in both control and sensing channels. However, the difference between the GFET responses from the control and sensing channels remains the same. Furthermore, we measured the GFETs devices response of different chips with various spiked model exosome concentrations from 10^–2^ μg/μL to 1 μg/μL in plasma. MCF-7 exosomes were selected as the model exosomes, since GPC-1 protein biomarker is also enriched on the surface of MCF-7 exosomes.^12,45−48^ (Figure S2). The calibration curve shows strong correlation (*y* = 9.92 log(*x*) + 44.67, *R*^2^ = 0.9990 and SD = 0.1700) between concentration and GFETs response when internal control is used while weaker correlation with higher standard deviation (*y* = 17.21 log(*x*) + 65.27, *R*^2^ = 0.9311 and SD = 1.3148) is observed without the use of control measurements (Figure S3). The graphene surface directly interacts with the buffer solution, and ions can diffuse and accumulate at the graphene surface in addition to multiple interactions at the graphene/buffer interface, which can result in the drift in the Dirac voltage. As both sensing and control channels undergo the same conditions, therefore the signal difference between sensing and control channels can significantly reduce the error resulting from the drift and increase the reliability of the detection.

In addition, one of the critical aspects of a biosensor is device uniformity and reproducibility. The uniformity and reproducibility of the GFET sensor performance were investigated by comparing the GFETs response for five chips to the same concentration of spiked target exosomes (10 ng/μL) in buffer. The change in Dirac voltage remains at 13.48 ± 0.71 mV with 5% chip to chip variation as shown in Figure 2D. Furthermore, the GFET devices on each chip show a small device-to-device variation. The average change in the Dirac voltage was within 2%. Therefore, we found the repeatability and the uniformity in electrical response from chip-to-chip and device-to-device are satisfactory for clinical testing.

Moreover, high efficiency in capturing exosomes on the sensor surface is crucial to achieving high sensitivity of GFETs. PBASE is widely used as a linker molecule to immobilize antibodies on graphene due to its ester function.^38−41^ However, the PBASE molecule has a highly flexible alkyl end chain that may lead to multiple orientations of antibodies on the graphene surfaces. Therefore, we proposed using a tetrakis(4-carboxyphenyl) porphyrin (TCPP) for functionalization and antibody immobilization on the surface of our GFET devices. The bulky TCPP molecules are more stable and can result in a more controlled proper orientation of antibodies on graphene. We compared the GFETs performance after immobilization of CD63 antibodies on graphene surface functionalized with TCPP and PBASE. We found up to 3-fold improvement in GFETs response to exosome (0.01 μg/ μL) when the surface was modified with TCPP compared with PBASE. The enhancement in response for TCPP devices was correlated with the increase in the number of exosomes captured on graphene surface compared with PBASE (see Materials and Methods and Figure S4 for further information).

We also implemented an optimized test protocol that includes separated incubation and testing steps. Tests of clinical samples included multiple rinsing and electrical testing steps prior to the injection of undiluted plasma samples onto the GFET chip. As the GFET biosensor’s response is strongly affected by the ionic strength of the solution, the chips were rinsed with PBS after 15 min of incubation of the samples. In order to remove the false positive signals arise from the highly viscous plasma residue on the surface of graphene, the amount and number of ×1000 diluted PBS used to rinse after the sample incubation step is standardized to be 3× times of 200 μL of PBS each time. (Figure S5) All measurements were recorded in ×1000 diluted PBS in order to reduce the screening effect. This should lead to an improved sensing performance and reliability of the sensor response.

Furthermore, in order to ensure the stability and reliability of the GFET devices during clinical testing, we set up device exclusion criteria that enable highly reproducible and reliable results to be collected based on more than 440 laboratory-tested devices (Figure 2E). The Dirac voltage for the lab-tested devices was *V*_D_ = 0.71 ± 0.24 V. To eliminate the effect of device drift, devices with *V*_D_ < 0.47 V or *V*_D_ > 0.95 V were excluded in this study. The average resistance of the devices was measured in air at *R* = 1230 ± 400 Ω, and therefore devices that exhibited values of *R* > 1700 Ω or *R* < 700 Ω were also excluded.

To evaluate the analytical performance of developed GFETs, we performed transfer characteristic measurements on GFET sensors for the detection of spiked model exosomes in buffer. The characteristic *I*–*V* curves of the GFET biosensor upon detection of exosomes in PBS in concentrations from 10^–1^ ng/μL to 10^3^ ng/μL were recorded. A significant left shift of the Dirac point with increased exosome concentration in comparison to that of the buffer solution is shown (Figure S6). Figure 2F shows the calibration curve of the change in Dirac voltage against target concentration (*y* = 4.06 log(*x*) + 9.68; *R*^2^ = 0.9823). A logarithmic dependence of spiked exosome concentration with the device shift is shown. This proves the excellent sensitivity of the GFET sensors.

The selection of the antibody is also crucial in achieving the high selectivity and sensitivity of biosensors. Many exosome-enriched proteins have been reported. Specific exosomal enriched proteins are expressed more on pancreatic cancer cell-derived exosomes compared to healthy ones, which offers the possibility to diagnose and distinguish patients with PC.^12−14^ Protein biomarkers enriched on pancreatic cancer-cell-derived-exosomes include glypican-1 (GPC-1),^12^ RHOB,^49^ CD63 and Prom1.^50^ Therefore, the selectivity of each protein biomarker to cancer cell-derived compared to healthy exosomes was tested. Four types of antibodies (CD63, GPC-1, Prom1, and RhoB) were immobilized on the graphene surface to investigate their sensitivity and selectivity to PDAC cancer exosomes in patient plasma samples (see Section S2 for details of the functionalization protocol for the graphene surface). Figure S8 summarizes the performance of these antibodies in differentiating PDAC cancer exosomes from healthy exosomes in the blood plasma. GPC-1 Ab (antibody) was used as a putative cancer marker for the detection of cancerous exosomes using the GFET biosensor; it was selected to be used for conjugation onto the GFET surface in the clinical testing (Section S3 and Figure S8).

In order to test the selectivity of GFETs for GPC-1+ cancerous exosomes prior to clinical testing, we spiked healthy plasma samples with model exosomes and measured the GFET responses with and without spiked exosomes. The measurement results are shown in Figure 2G. The healthy control plasma induced a small signal of 3.5 ± 2.7 mV, which could be attributed to the small percentage of GPC-1 present in the healthy plasma. There are around 0.3–4.7% GPC-1+ exosomes in healthy human plasma samples.^48^ On the other hand, the addition of various concentrations of model cancerous exosomes (0.01 μg/μL, 0.1 μg/μL and 1 μg/μL) caused a significant shift in Dirac voltage of 37 ± 7.9 mV. This indicates that our GFET biosensor can detect cancerous exosomes in blood plasma without any processing or preparation required. The detection results also demonstrate the high selectivity of the GFET sensor to cancerous cells in comparison to healthy exosomes.

### Clinical Detection of PDAC Cancer Patient Samples with GFET

**Figure 3:**
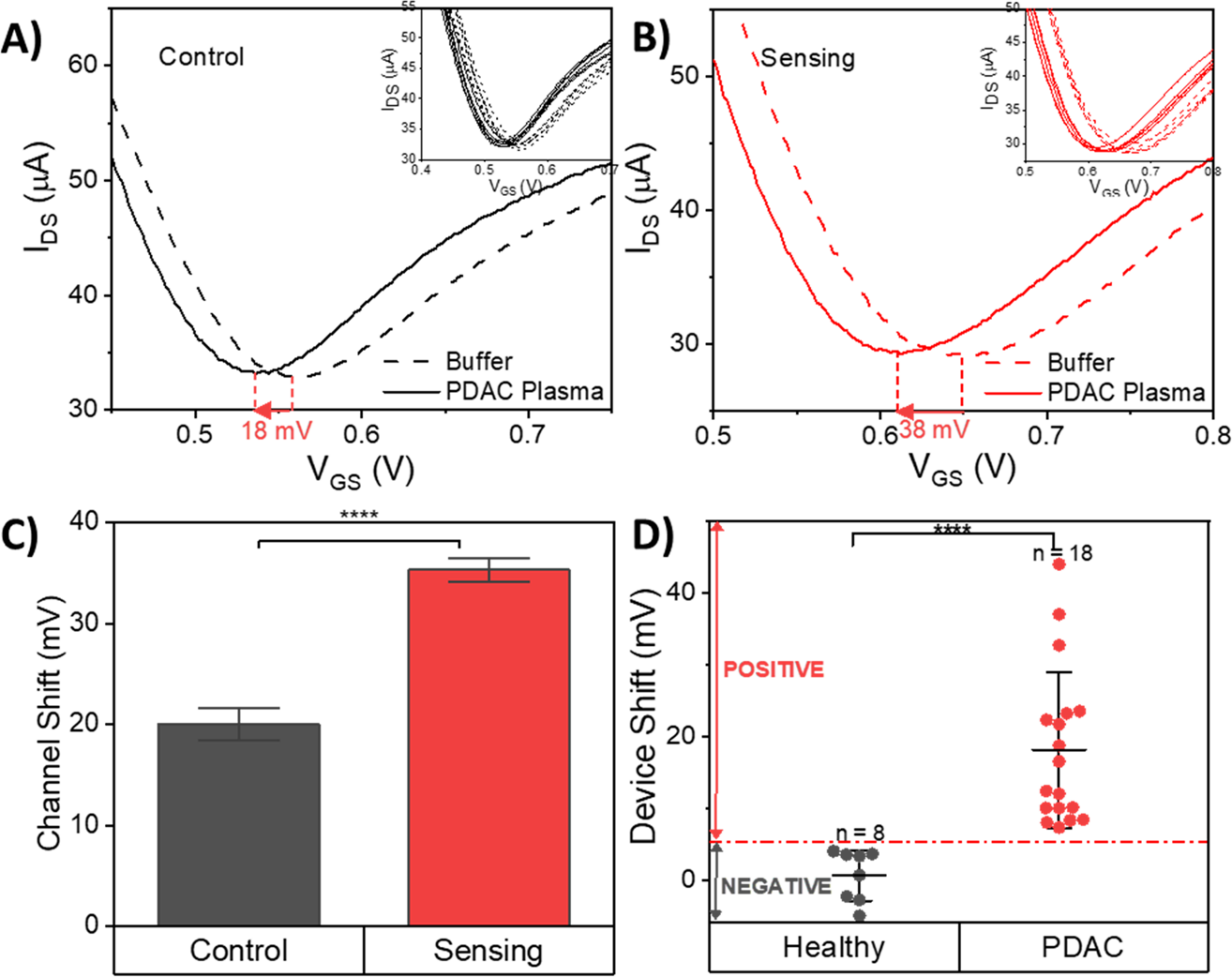
Clinical detection of PDAC cancer exosomes in PDAC patient and healthy control plasma using the GFET biosensor. (A) Representative *I*_DS_–*V*_GS_ curve of one of the control channels on the GFET biosensor before and after PDAC plasma incubation. The inset image shows the response from 6 control channels on the GFET biosensor. (B) Representative *I*_DS_–*V*_GS_ curve of one of the sensing channels on the GFET biosensor before and after PDAC plasma incubation, showing a significant curve shift in the sensing channel. The inset image shows the response of 6 sensing channels on the GFET biosensor. (C) Device shift of the 6 control and 6 sensing channels on a representative clinically tested GFET device. The device shift arising from a plasma sample is calculated based on the difference between the GFET response from control and sensing channels. (D) Measurement results for the GFET biosensor from the clinical testing with plasma samples from 18 PDAC patients and 8 healthy controls, indicating a clear threshold detection signal line, which suggests that the sensor is able to differentiate PDAC patients from healthy controls (****P< 0.0001). Data are mean ± standard deviation (s.d.).

Next, we used the developed GFET biosensor for the detection of GPC-1+ exosomes in blood plasma with a cohort of 26 patients, including 18 cases of PDAC patients and 8 healthy controls (Table S1 for a summary of the patient cohort). Only a small droplet (20 μL) of each sample is required for testing with a GFET sensor. We used the internal control channel and BSA to reduce nonspecific binding and false positives (Figure 3A). GFETs transfer curves in buffer solution inherently drift due to several possible reasons, including ion redistribution after the application of an electric field, trapped ions between graphene and substrate, defects in graphene or interface traps in the substrate that can generate a false positive response and reduce the accuracy of detection.^51,52^ Therefore, utilizing internal control is crucial to compensate and minimize the effects of the drifts and reduce the background noise.^41^Figure 3B shows a representative graph of the GFET transfer curve before and after the incubation of a patient sample on the surface of graphene. The difference in the Dirac voltage shift between sensing and control channels was used to determine the GFET response. The measurements before and after incubation were taken in ×1000 diluted PBS to ensure strong coupling between the graphene and exosome charges in the electrical double layer. The presence of 6 GFETs in the internal control and 6 in the sensing channels improves the statistical analysis and reduces the detection error. Figure 3A and B shows a representative response from the total of 12 channels, including 6 control and 6 sensing channels. Highly uniform and small device-to-device variations in response are usually observed (see the inset in Figure 3B). The measurement results from the 26 patient samples are shown in Figure 3D. Of all the devices tested, 281 GFETs satisfied the exclusion criteria, and only the test results from these devices were included in the clinical testing (Figure S9). Our GFET biosensor can clearly discriminate between PDAC patients and healthy controls, with a clear threshold line at 4.5 mV (Figure S10). There is an average shift in Dirac voltage with healthy control plasma of 0.5 ± 3.5 mV (mean ± s.d.), whereas all PDAC patients showed a significant change in the Dirac point larger than 7.3 mV with an average shift of 18.0 ± 10.9 mV (mean ± s.d.). This increase in GFET response could be a result of higher levels of GPC-1+ exosomes produced by tumorous cells in the PDAC patients’ blood plasma.^48^

### Validation of GFET Response to GPC-1 Exosome Concentration

**Figure 4:**
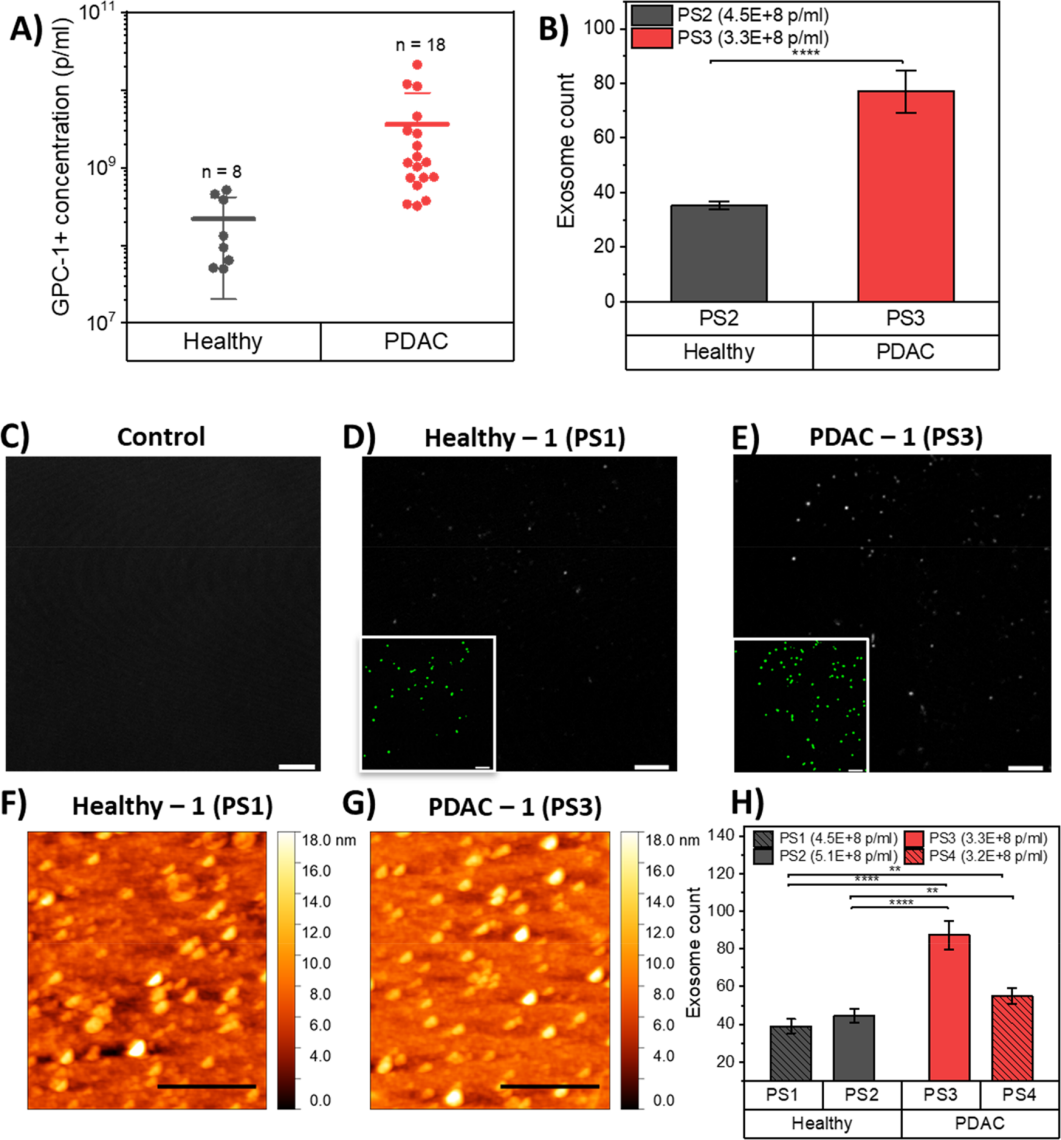
Validation of the detection of PDAC cancer exosomes in PDAC patient and healthy control plasma. (A) Concentrations of GPC-1+ exosomes detected in 18 PDAC patient plasma samples and 8 healthy control plasma samples by Nano Flow cytometry measurements. Comparison of the numbers of PDAC and healthy exosomes captured on GFET biosensors. (B) Fluorescence confocal microscopy images of exosome count on GFET sensors taken from an average of three positions on the functionalized graphene surface incubated with PDAC plasma sample and three positions incubated with healthy plasma sample. (For details of the method used to count exosomes, see the Materials and Methods). (****P < 0.0001). (C) Functionalized graphene surface with no exosome as control. (D) Functionalized graphene surface incubated with healthy plasma sample (PS1). (E) Functionalized graphene surface incubated with PDAC plasma sample (PS3). Exosome signal is shown in gray scale, and the inset shows the same field of view with the detected exosomes after bioimage analysis in green to make it clearer. Confocal scale bars = 5 μm. (F) Atomic force microscopy images of functionalized graphene surface incubated with healthy plasma sample (PS1). (G) AFM images of functionalized graphene surface incubated with PDAC plasma sample (PS3). (H) Exosome count on GFET sensors taken from an average of eight spots on each of the functionalized graphene surfaces incubated with healthy plasma samples (PS1 and PS2) and eight spots on incubated with PDAC plasma samples (PS3 and PS4). (**P < 0.01, ****P < 0.0001). AFM scale bars = 1 μm. Data are mean ± s.d.

**Figure 5:**
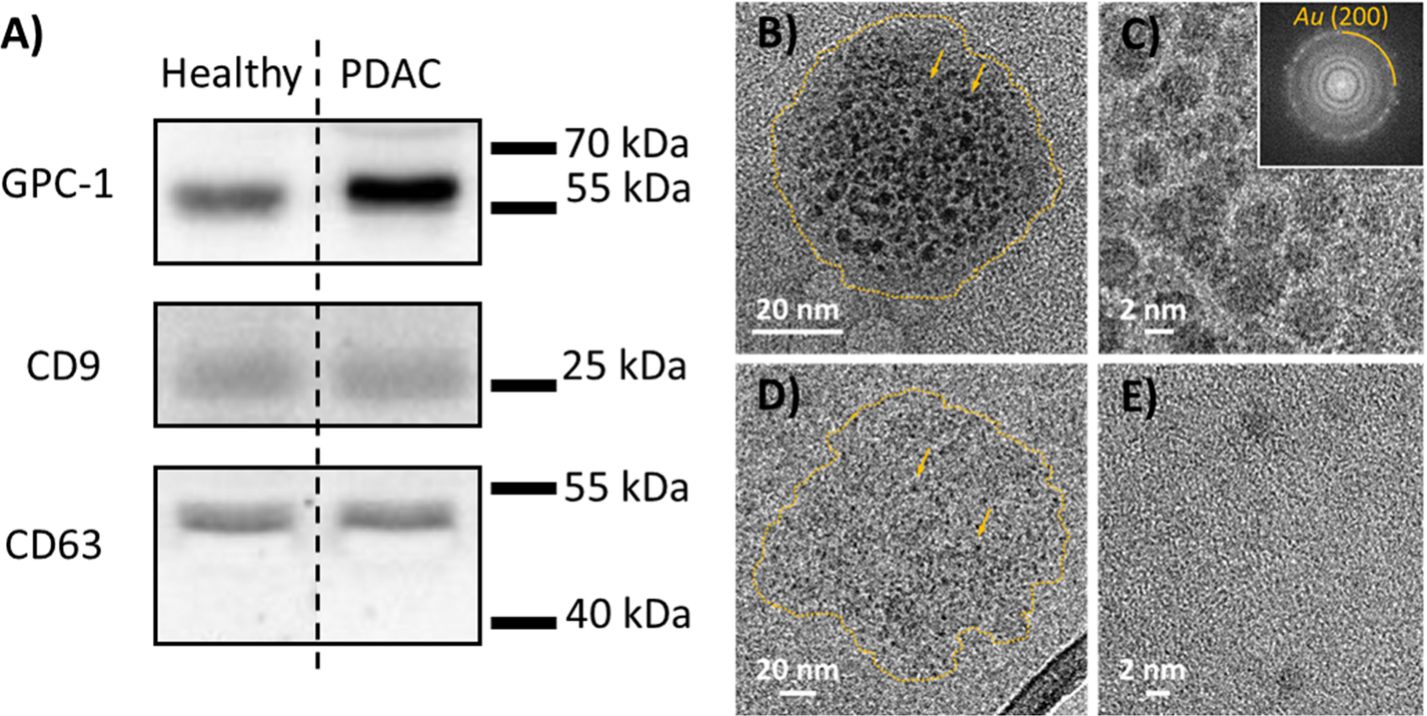
Western blot and IG-TEM validation of GPC-1 expression in PDAC cancerous and healthy exosomes. (A) Western blot of GPC-1, CD9, and CD63 in exosomes extracted from healthy plasma and PDAC patient plasma. (B) Low-magnification IG-TEM image of GPC-1 in PDAC cancer exosomes with immunogold labeling. The outlines of individual exosomes are highlighted by a dashed yellow line for clarity. (C) High-resolution IG-TEM image of GPC-1 on the surface of a PDAC cancer exosome with immunogold labeling. Inset shows the respective FFT pattern with the prominent ring corresponding to the (200) planes of Au. (D) Low-magnification IG-TEM image of a healthy exosome with immunogold labeling. The yellow circles around individual exosomes are given for visual guidance. The arrows point to the individual Au particles on the surface of a healthy exosome. (E) High-resolution IG-TEM image of GPC-1 on the surface of a healthy exosome with immunogold labeling.

In order to further validate the results, we performed Nano Flow cytometry measurements,^53,54^ a commonly used power analytical tool for biological nanoparticles like exosomes down to less than 100 nm diameter by light scatter, to determine the concentrations of GPC-1+ exosomes in the plasma samples from the healthy controls and PDAC patients tested. Figure 4A shows that GPC-1+ exosome concentrations were significantly higher in patient samples than in healthy ones, with average levels of GPC-1 found to be an order of magnitude higher in the former (range from 3.21 × 10^8^ to 2.11 × 10^10^ particles/mL, with a mean of 3.59 × 10^9^ particles/mL) compared to the latter (range from 4.96 × 10^7^ to 5.13 × 10^8^ particles/mL, with a mean of 2.18 × 10^8^ particles/mL). Our data are in close agreement with that previously published in the literature,^48^ indicating that the expression of GPC-1+ exosomes in blood plasma significantly increases in tumor-derived exosomes compared to healthy normal cells. Interestingly, we found three PDAC samples with concentrations of exosomes at levels similar to those in healthy samples, while the GFET responses were found to be twice as strong as for the healthy ones. Compared with healthy ones, cancerous exosomes have generally been reported to carry more electrical charge at their surfaces, and this could contribute to the increase in the GFET response for PDAC samples.^55^ However, this may not explain the significant increase in the sensor response for the PDAC samples. Another possible explanation could be a high binding affinity between GPC-1+ and antibodies, which could result in a higher density of exosomes on the surface. Therefore, we performed two fluorescence confocal microscopy experiments on PDAC samples with a concentration of exosomes of 3.38 × 10^8^ particles/mL and healthy samples with an exosome concentration level of 4.54 × 10^8^ particles/mL. We found that the exosome density on the graphene surface was 54.5% higher for PDAC samples compared to healthy samples (Figure 4B, D, E). In order to further validate this finding and to quantify the number of particles on the surface, we used other independent techniques, AFM, to image the number of particles on the surface (Figure 4F, G, H) and SEM (Figure S11) on 2 PDAC and 2 healthy samples with similar levels of exosome concentration in the plasma. The results were found to be consistent with those from fluorescence imaging. Based on scanned areas of 800 μm^2^ for AFM and 100 μm^2^ for SEM for each sample, we found an average of 55.3% and 58.6% times higher percentage of PDAC exosomes on the graphene surface compared with healthy exosomes. In the measurements using Nano Flow cytometry, the exosomes in healthy control and PDAC patient samples have similar size distributions (80.0 ± 5.8 nm for PDAC exosomes; 85.7 ± 6.7 nm for healthy exosomes). Therefore, diffusion does not play a role in the enhancement of PDAC capture on the sensor surface. The higher response from the GFET sensor to PDAC samples could be due to higher GPC-1 expression on exosomes from PDAC plasma samples compared to those from heathy plasma samples,^12,56^ leading to a higher binding affinity between the antibody and PDAC exosomes. Therefore, we isolated exosomes from PDAC patient blood plasma and healthy control blood plasma (Section S4 and Figure S12) Western blot analysis was performed to examine the isolation and expression of GPC-1 proteins on isolated plasma exosomes (Figure 5A and Figure S13). We detected an elevated level of both CD9 and CD63 as exosomal markers^57,58^ (Figure S13). These results indicate that the exosomes were successfully isolated from patient and healthy control plasma samples. Furthermore, the Western blot results suggest that the exosomal GPC-1 protein levels in the PDAC patient samples were significantly higher than those in healthy control samples (Figure 5A). This indicates a much higher level of GPC-1 protein expression on PDAC exosomes. To further validate the high expression of GPC-1 protein on PDAC exosome, we performed TEM imaging with immunogold labeling to directly reveal the GPC-1 protein density on the surface of healthy and cancerous exosomes (Materials and Methods). TEM with immunogold labeling identified large number of GPC-1 proteins are exhibited on the surface of cancerous exosomes while very small number of GPC-1 proteins are presented on the surface of healthy exosomes (Figure 5B, C, D, E, and Figure S13). The multiple GPC-1 proteins on the surface of the cancerous exosomes can increase the accessibility of the exosomes to the antibodies on the surface by increasing the number of proteins expressed on the exosome surface. Furthermore, the presence of multiple available sites on the exosome surface can promote cooperative reactions and increase the vividity and affinity to the antibody.^59^ For example, having many specific proteins on the surface of the exosome could allow several adjacent antibodies to bind more effectively to the exosome. This could explain the higher density of cancerous exosomes on the graphene surface relative to the healthy ones and a higher response from our GFET sensor to samples from PDAC patients than healthy controls, especially given that the numbers of exosomes present in the plasma were the same for both samples.

It is worth noting that there are many free circulating GPC-1 protein in the blood plasma and may lead to small false positive signals of the GFET biosensor. It is reported that the concentration of GPC-1 protein in plasma is small compared to circulating exosomes, generally between 8.74–32.67 ng/mL.^60^ Meanwhile, the GPC-1 protein concentration should be observed in both healthy controls and PDAC cancer plasma samples. In our previous work,^61^ we found there is no significant difference between the level of circulating GPC-1 proteins in healthy controls and PDAC patients’ plasma.^61^ During our clinical detection, we observed a small shift in the device response from healthy samples, which is the sum of the GPC-1 protein + GPC-1+ exosome. These indicate that the contribution of the GPC-1 protein is negligible in comparison to that of the GPC-1+ exosome.

### Assessment of the GFET Response versus the Stage of Cancer

**Figure 6:**
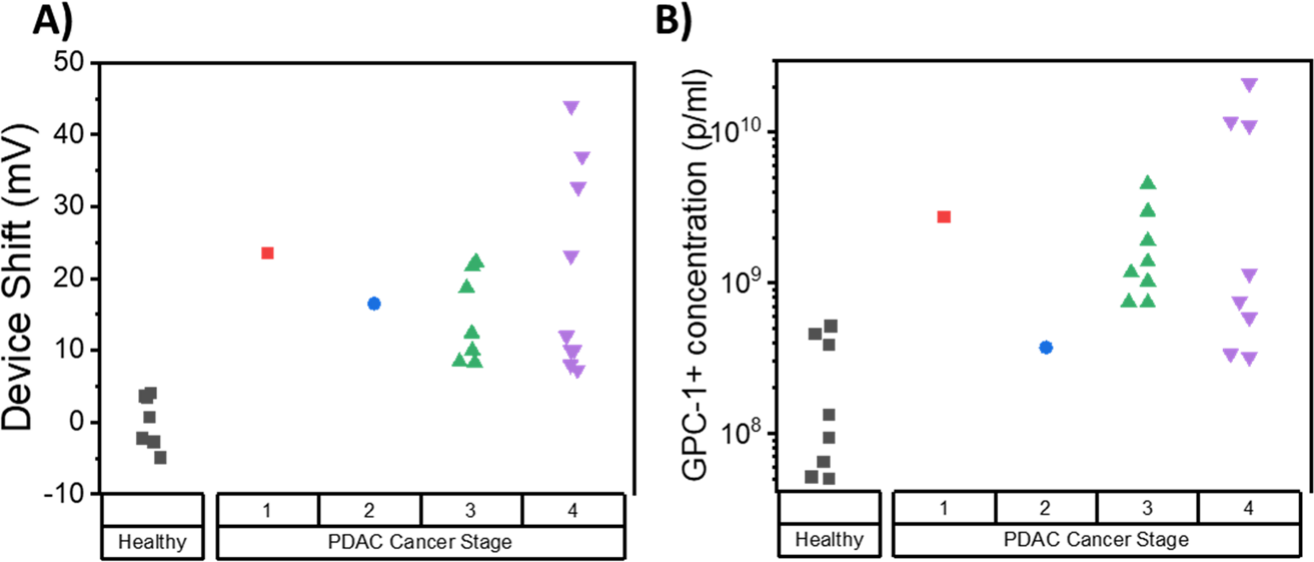
Detection of all PDAC cancer stages with a GFET biosensor. Measurement results of PDAC cancer stages, including stage 1, 2, 3, and 4, were classified using (A) detection signals from the GFET biosensor and (B) GPC-1+ particle concentrations from Nano Flow cytometry measurements.

Finally, we summarized the GFET signal with respect to the stage of PDAC cancer obtained from CT and MRI. The difference in the change of Dirac voltage of the GFET sensor as a function of the cancer stage is plotted in Figure 6. Interestingly, the results indicate that the GFET biosensor detected all stages of PDAC cancer, including early stage 1. However, the result shows no significant correlation between the signal of the GFET sensor and the stage of cancer. Moreover, there is no significant correlation between the concentration of exosomes in patient plasma and the stage of cancer (*P* = 0.223), which is consistent with previous findings in the literature. The independence of exosome concentration and PDAC stage indicates that our GFET biosensor technology could have high potential for use to detect PDAC cancer even at a very early stage of the condition.

## Conclusion

Pancreatic cancer (PC) is known for difficult early stage detection and poor survival prognosis, and patients present for medical evaluation only when the cancer is advanced and they experience signs and symptoms. Early detection can significantly improve the survival rate and outcome. In this study, we proposed an electrical test for the detection of cancerous exosomes using a GFET platform as a promising route for the early detection of pancreatic cancer. The test can be performed on whole plasma samples using a drop of 20 μL with a total detection time of less than 45 min, which can be greatly reduced by using a faster readout electronic system. The test can detect all stages of cancer, taking advantage of the characteristics of cancer exosomes and the high sensitivity of GFETs. Based on samples from 26 patients, we found that cancer exosomes such as GPC-1 exosomes are present in much higher levels in PDAC patient samples than in healthy ones. Although there was an overlap in the levels of exosomes in some samples, we found that the density of exosomes on the GFET surface and the GFET response are significantly higher for cancer patient samples than those from healthy controls. After analysis of the GPC-1+ exosomes in pancreatic cancer and healthy plasma using Western blot analysis and IG-TEM, we found that GPC-1 proteins are more strongly expressed on pancreatic cancer exosomes compared to healthy ones. This could play a crucial role in the binding affinity and enables GPC-1 to have higher specificity to cancer exosomes than healthy ones; therefore, it increases the induced signal in the GFETs biosensors and improves the accuracy of detection. This high expression of charged proteins on the GPC-1+ cancer exosomes makes GFETs sensors suitable for accurate discrimination between cancer and healthy samples, which is otherwise challenging when other methods such as ELISA or Nanoflow are used particularly when the healthy and cancer plasma samples have the same level of GPC-1 exosome. The portable platform with read-in/read-out electronics makes the test simple and user-friendly, with no need for a trained professional to perform it. The presence of internal control on the GFET chip has significantly reduced false positive rates and improved the accuracy of detection. The test procedure consists of four simple steps including two blank measurements, incubation, and multiple rinsing, which does not rely on skilled operator. Our GFET platform is adaptable and can be used to detect multiple pancreatic cancer biomarkers simultaneously on the same chip. Beyond PDAC detection, this GFET technology can be reconfigured to facilitate the detection of other disease biomarkers, which could be crucial for diagnosis purposes. Moreover, the GFET chip is CMOS compatible and can be manufactured on a large scale, which can massively reduce costs with small device-to-device variation and a high yield.

## Materials and Methods

### Fabrication of On-Chip Integrated Graphene Field Effect Transistor Sensor Array

The GFET sensor array was designed with on-chip liquid gold (Au) gate electrodes and manufactured on 4” and 6” wafers in the Graphenea foundry (https://www.graphenea.com/). The steps in manufacturing include CVD graphene growth on Cu foil, graphene transfer onto Si/SiO_2_ substrate, graphene patterning using a combination of photolithography and O_2_ plasma etching, Au metallization, device passivation with Al_2_O_3_ deposited using atomic layer deposition, and then chip dicing. The carrier mobilities of individual GFETs, as determined from the transfer curves and applying back gates, are on average 1700 cm^2^ V^–1^ s^–1^ with a standard deviation of σ = 280 cm^2^ V^–1^ s^–1^ determined for 10 batches of devices. The average yield for each wafer is >95% according to quality control measurements on a larger number (*n* = 310) of chips.

### Functionalization of Antibody on GFET Biosensor

The biofunctionalization step mainly includes two steps of incubation with the linker molecule tetrakis(4-carboxyphenyl) porphyrin (TCPP) that can bind to graphene via an π–π interaction, followed by a step of antibody conjugation through the formation of covalent amide bonds with the linker molecules. First, the GFET devices were incubated with TCPP (50 μM) (Tokyo Chemical Industry) in 2-methoxyethanol (Tokyo Chemical Industry) for 2 h at room temperature before being rinsed with 2-methoxyethanol and 1 × PBS to remove excessive TCPP from the graphene surface and then dried with N_2_. The carboxyl groups on TCPP were activated via EDC/NHS for 30 min at room temperature with a mixture of 200 mM 1-ethyl-3-(3-(dimethylamino)propyl) carbodiimide hydrochloride (EDC·HCl) (Sigma-Aldrich) and 50 mM *N*-hydroxysuccinimide (NHS) (Sigma-Aldrich) in DI water. Then, samples were rinsed with 1 × PBS and DI water and dried with N_2_. Then, the anti-CD63 antibody (BD Bioscience US) or anti-GPC-1 antibody (Invitrogen PA5–28055) were used. The antibodies were supplied in a stock solution of 0.5 mg/mL in an aqueous buffer solution (containing ≤0.09% sodium azide) and were diluted using 1 × PBS to a concentration of 100 μg/mL. Droplets of 20 μL of 100 μg/mL anti-CD63 antibody or anti-GPC-1 antibody were placed on the surface and left overnight in a humidified environment at 4 °C. The samples were then sequentially rinsed with 1 × PBS and DI water and dried with N_2_. Samples were blocked using 1% bovine serum albumin (BSA) in 1 × PBS at room temperature for 1 h, rinsed with PBS and DI water, and dried with N_2_. The prepared samples were stored at 4 °C for later use.

### Functionalization with PBASE

Samples were first incubated for 2 h with 3 mM PBASE (Sigma-Aldrich) in dimethylformamide (DMF) (Sigma-Aldrich) at room temperature. Then DMF and DI water were used to gently rinse the GFET sample to remove excessive PBASE from the surface and dried with N_2_. Then, the anti-CD63 antibody (BD Bioscience US) were used. The antibodies were supplied in a stock solution of 0.5 mg/mL in an aqueous buffer solution (containing ≤0.09% sodium azide) and were diluted using 1 × PBS to a concentration of 100 μg/mL. Droplets of 20 μL of 100 μg/mL anti-CD63 antibody were placed on the surface and left overnight in a humidified environment at 4 °C. The samples were then sequentially rinsed with 1 × PBS and DI water, and dried with N_2_.

### Graphene Characterization Techniques

#### AFM

AFM was performed using an Asylum MFP-3D classic and a Bruker Innova system in AC tapping mode with SCOUT 70 tips of average radius 15 nm and typical scan resolution of 512 pixels × 512 pixels. All AFM scans were performed under dry conditions.

For the quantitative analysis of each sample, 3 to 5 images were recorded and processed using Gwyddion image analysis software.^62^ Images obtained were analyzed using ImageJ^63^ to obtain the density of particles on the graphene surface. Images were processed using a color threshold with the threshold color set as B&W and color space as RGB, with the same threshold value applied. Then, the particles were counted using the “analyze particles” toolbox in ImageJ.

#### XPS

XPS experiments and measurements were performed with K-Alpha+ and an Al radiation source (*hv* = 1486.6 eV) in an ultrahigh vacuum chamber for spectroscopic analysis with a base pressure of 5 × 10^–8^ mbar.

#### Raman Spectroscopy

Raman spectroscopy measurements were performed using a LabRAM HR Evolution Raman spectrometer (Horiba Scientific) and excited with laser (Torus MPC 3000) with a wavelength of 532 nm (excitation energy *E*_L_ = *h*ω_L_ = 2.33 e) through an optical fiber, with an objective lens of 100×, NA = 0.8, and a laser spot of 0.4 μ. The laser power was kept below 2 mW and the diffraction grating was 600 mm/groove. The range of the Raman spectra collected spanned the wavenumber region 1200–3000 cm^–1^. The Raman peak position was calibrated based on the first order Raman signal of silicon, at 520.7 cm^–1^.

#### Scanning Electron Microscopy

Scanning electron microscopy (SEM) imaging was performed under secondary electron mode by using a Zeiss Leo (1525) system with an accelerating voltage of 5 kV. A normal operating vacuum of 2 × 10^–5^ mbar was achieved during the pump down of the chamber. The graphene on the substrate was mounted on the metallic sample holder using carbon tape. The samples were coated with 15 nm chromium using a turbomolecular pumped sputter coater (EMS150T Plus) before performing SEM microscopy. Images obtained were analyzed using ImageJ,^63^ as described above, for particle size and density analysis.

#### Confocal Microscopy

Sample immunofluorescent labeling: After surface functionalization with TCPP and activation with EDC/NHS at room temperature, the sample surface was then conjugated with 5 μL of 0.33 mg/mL anti-GPC-1 antibodies (Invitrogen PA5–28055) overnight at 4 °C in a humidified environment. After antibody conjugation, the samples were rinsed with 1 × PBS and DI water, and dried with N_2_. The surface was then blocked using 1% bovine serum albumin (BSA) in 1 × PBS at room temperature for 1 h, rinsed with PBS and DI water, and dried with N_2_. Next, 20 μL of blood plasma samples were added to the surface and incubated for 0.5 h. The samples were then rinsed and labeled with Alexa Fluor 647 anti-GPC-1 antibodies (Abcam, ab237290) (20 μL, 1:100 diluted in 1 × PBS) at room temperature for 1 h in the dark. The samples were then rinsed with 1000 × PBS and DI water, and dried with N_2_. For confocal microscopy, surfaces were placed upside-down in a 35 mm Ibidi imaging dish and imaged on a Leica SP8 point scanning microscope with a 63×/1.4 NA Plan Apo objective, with a zoom of 4 and 1024 × 1024 pixels (px) per frame, giving a xy pixel size of 45 nm. Exosomes were labeled with Alexa Fluor 647 anti-GPC-1 antibodies (Abcam, ab237290) and excited with a 633 nm laser. Fluorescence was detected on a HyD detector between 643 and 782 nm.

Sixteen bit LIF files were opened in Icy^64^ and exosomes were detected using the Spot Detector plugin,^65^ with a minimum size of 10 px. A rectangular ROI of 240 × 120 px was drawn in the field of view in a region without detection, and the mean fluorescence intensity was calculated. Only detection cases with a mean fluorescence intensity of at least twice the background intensity were counted.

### Nano-Flow Cytometry

Plasma GPC-1+ exosome concentration and sizing were measured by Nano Flow cytometry using a NanoAnalyzer U30 instrument from NanoFCM Inc. (Nottingham, UK). First, 50 μL of each blood plasma sample was incubated with 1 μL of Alexa Fluor 647 conjugated anti-Glypican antibody (Abcam, ab237290) for 30 min at room temperature. After incubation, the mixture was diluted in PBS to 1 mL to pellet EVs, followed by ultracentrifugation at 55,000 rpm (100,000*g*) with a benchtop optima TLX (Beckman Coulter) for 45 min. The supernatant was removed, and the pellet was resuspended in 50 μL of PBS. The resuspended mixture was analyzed in the Flow NanoAnalyzer to determine concentration and fluorescence positivity. Data processing was performed by using nFCM Professional Suite v1.8 software.

### Exosome Isolation from Human Plasma Samples

Izon qEV Original columns (70 nm separation) were used. Columns were rinsed with 10 mL of PBS, then 1 mL of plasma sample was loaded to the center of the qEV column, followed by elution with PBS. Fractions 7–10 were collected as EV samples; 2 mL of the isolated EV samples were collected.

### Western Blot Analyses

Isolated exosomes were concentrated using Vivaspin Turbo 4 (Vivaspin Turbo 4, 10 kDa, PES, Sartorius) at 4000*g* for 2 h at 4 °C. Cell lysis RIPA Buffer (No. 9803, Cell Signaling Technology) and additive PMSF (200 mM, Cell Signaling Technology) were used. The EVs were lysed with PMSF at 1:200 (final concentration 1 mM) and RIPA 10× at a ratio of 1:10. The EVs and lysis buffer were vortexed for 20 s and lysed on ice for 30 min. Then the samples were centrifuged at 12,000*g* for 20 min. The supernatant was transferred to a new tube. The concentration of EVs was measured using the Pierce BCA Protein Assay Kit (Thermo Scientific). The Western blot analyses were then performed in DTT and Sample Buffer (Pierce LDS Sample Buffer, Non-Reducing (4×), Thermo Scientific) using the following antibodies: 1:500 ALIX (#92880, Cell Signaling Technologies), 1:250 Glypican-1 (PA5–24972, Thermo Scientific), CD9 (sc-13118, Santa Cruz, mouse), CD63 (MX-49.129.5, Santa Cruz).

### Immunogold Transmission Electron Microscopy

Transmission electron microscopy (TEM) characterization was performed using a JEOL JEM-2100F field emission S/TEM equipped with an Oxford X-MaxN 80 mm2 SDD detector for elemental analysis. TEM images were acquired at a 200 kV accelerating voltage and 116 μA emission current. DigitalMicrograph GMS3 (Gatan) and Aztec TEM (Oxford Instruments) software packages were used for the TEM data and energy dispersive X-ray (EDX) spectral analysis and interpretation, respectively. Briefly, for the immunogold labeling with antibodies, GPC-1 antibodies (pa5–51290, Thermo Scientific) were attached on 10 nm gold particles (AURION) according to the manufacturer’s instructions at room temperature. Healthy plasma and PDAC plasma sample pools were prepared. 200 μL from each of 5 healthy or 5 PDAC plasma samples was taken to form a healthy plasma or PDAC plasma sample pool. Exosomes were isolated as previously described. Then isolated exosome samples were conjugated with the gold-particles attached GPC-1 antibodies at the appropriate dilution overnight at 4 °C. Samples for TEM characterization were drop-cast from dilute aqueous suspensions onto amorphous carbon coated (200 nm) Cu grids (Agar Scientific) and dried naturally overnight.

### Clinical Samples

Patient samples were obtained through the *CIRcular and Noncoding RNAs as Clinically USeful Biomarkers in Pancreaticobiliary Cancers* (CIRCUS) clinical trial at Royal Surrey County Hospital NHS Foundation Trust (NCT04584996). Research Ethics Committee (REC) approved, IRAS Project ID: 277406.

All patients planned for surgical resection for PDAC were identified through the HPB multidisciplinary team (MDT) meeting and by the clinical team at Royal Surrey County Hospital NHS Foundation Trust. These patients were approached either in clinic or at the preoperative assessment. Plasma samples were taken from resectable PDAC patients and those with locally advanced, borderline, or metastatic PDAC deemed unsuitable for surgical resection and for potential medical treatment (i.e., neo-adjuvant or palliative chemotherapy). The control group consisted of patients diagnosed and/or due to undergo surgery for benign pathology (e.g., gallstones, chronic pancreatitis, etc.)

Exclusion criteria for the patients are unwilling or unable to provide written informed consent, non-English speaking, known to be pregnant, aged <16 years, known diagnosis of HIV or Hepatitis B/C virus.

Blood samples (total 30 mL) were drawn into anticoagulant treated EDTA collection tubes, labeled, and placed on ice. Blood samples were centrifuged within 2 h of collection using a validated double spin operating method for plasma isolation by Oxford University Trust. Samples undergo 2 steps of spin. The samples were centrifuged at 800*g* at 21 °C for 15 min, followed by the second centrifugation at 4000*g* at 21 °C for 15 min. Following the centrifugation, samples were immediately transferred into clean aliquots and frozen at −80 °C.

### Electrical Measurements and Clinical Testing Protocol

Immediately following the functionalization and immobilization of the GFET biosensor, electrical measurements were performed in 0.001 × PBS (×1000 diluted PBS to ensure a solution with low ionic strength) using a portable electronic readout system.^41^ GPC-1 antibody was functionalized on the surface of the GFET biosensor for all of the electrical measurements. Source–drain voltage was fixed at 0.1 V, and the electrolyte gate was swept from 0.4 to 0.9 V at a sweeping rate of 2 mV/s (the reading time per channel is around 1 min), rendering source–drain currents in the order of tens of microamperes (μA) in ×1000 diluted PBS. Samples were tested using the developed on-chip GFET sensors with all characteristic I–V transfer curves recorded. For the laboratory investigation of the analytical performance of the GFET biosensor, MCF-7 exosomes were used as model exosomes, the working concentration of exosomes was prepared by 10-time serial dilution from the stock solution (MCF-7 exosomes (Abcam, ab239691)), and ×1000 diluted PBS was used as the solvent. For clinical detection with PDAC patient and healthy control plasma, the transfer curves in ×1000 dilution PBS buffer solutions were recorded first. Then, the plasma sample (20 μL) was added to the functionalized sensor surface for 15 min of incubation, followed by multiple wash steps with PBS and DI water. Finally, the sensor immobilized with exosomes in plasma was tested in buffer. Total testing time is 45 min that includes the 15 min incubation, multiple rinsing, and 24 min for two measurements.

### Statistical Analysis

For all experiments, quantitative results are presented as the mean ± standard deviation (s.d.), where *n* denotes the number of replications.

The statistical significance of the data was assessed using the two-sample Student’s *t* test and is designated with asterisks (*P < 0.05, **P < 0.01, ***P < 0.001 ****P < 0.0001).

## Supporting Information Available

Additional information and data: (i) Description of electronic readout system for electrical characterization; (ii) Detailed description and image for functionalization and characterization of graphene surface; (iii) Selection of antibody for PDAC cancer exosome detection; (iv) optical image of on-chip integrated GFET devices; (v) Validation of GFET biosensor for detection of exosome with the protocol of using control and sensing channel; (vi) Summary of patient cohort; (vii) Device exclusion criteria for GFET reproducibility and validity in clinical testing; (viii) Scanning electron microscopy analysis of exosomes from healthy controls and PDAC patient plasma samples captured on functionalized GFET sensors; (ix) Western blot and IG-TEM analysis of exosomes isolated from PDAC patients’ plasma and healthy plasma (PDF)
