# Supplementary material for: Graphene Sensor Arrays for Rapid and Accurate Detection of Pancreatic Cancer Exosomes in Patients’ Blood Plasma Samples
Source: ACS Nano. 2023 Jul 20;17(15):14619–31. doi: 10.1021/acsnano.3c01812 (PMC10416564; doi:10.1021/acsnano.3c01812)
Supplement: Supplementary file 1 — nn3c01812_si_001.pdf [file nn3c01812_si_001.pdf]

Supplementary Information

Graphene Sensor Arrays for Rapid and Accurate Detection of Pancreatic Cancer Exosomes in Patients' Blood Plasma Samples

Tianyi Yin[1,*], Lizhou Xu[1,2,*], Bruno Gil[3], Nabeel Merali[4,5,6], Maria S. Sokolikova[1], David C. A. Gaboriau[7], Daniel S. K. Liu[8,9], Ahmad Nizamuddin Muhammad Mustafa[1,10], Sarah Alodan[1], Michael Chen[1], Oihana Txoperena[11], María Arrastua[11], Juan Manuel Gomez[11], Nerea Ontoso[11], Marta Elicegui[11], Elias Torres[11], Danyang Li[12], Cecilia Mattevi[1], Adam E. Frampton[4,5,6,8], Long R. Jiao[8], Sami Ramadan[1,*], Norbert Klein[1]

1 Department of Materials, Imperial College London, London, SW7 2AZ, UK

2 ZJU-Hangzhou Global Scientific and Technological Innovation Center, Zhejiang University, Hangzhou 311200, China

3 Hamlyn Centre, Imperial College London, London, SW7 2AZ, UK

4 Oncology Section, Surrey Cancer Research Institute, Dept. of Clinical and Experimental Medicine, FHMS, University of Surrey, The Leggett Building, Daphne Jackson Road, Guildford, GU2 7WG, UK.

5 HPB Surgical Unit, Royal Surrey NHS Foundation Trust, Guildford, Surrey, GU2 7XX.

6 Minimal Access Therapy Training Unit (MATTU), University of Surrey, The Leggett Building, Daphne Jackson Road, Guildford GU2 7WG, UK.

7 Facility for Imaging By Light Microscopy, Imperial College London, London, SW7 2AZ, United Kingdom

8 Dept. of Surgery & Cancer, Imperial College London, Hammersmith Hospital campus, London W12 0NN, UK

9 HPB Surgical Unit, Imperial College Healthcare NHS Trust, Hammersmith Hospital, London, W12 0HS, UK

10 FTKEE, Universiti Teknikal Malaysia Melaka, 76100 Durian Tunggal, Melaka, Malaysia

11 Graphenea Semiconductor, Paseo Mikeletegi 83, San Sebastián, ES 20009, Spain

12 Research Center, The Seventh Affiliated Hospital, Sun Yat-sen University, Shenzhen 518107, China

*Email: tianyi.yin17@imperial.ac.uk.

*Email: lzxu@zju.edu.cn.

*Email: s.ramadan@imperial.ac.uk.

Supplementary Results

S 1 Electronic readout system

Electrical characterization of the different graphene sensing units contained inside the GFET chip array was performed using a custom-designed and portable readout system with a dedicated graphical user interface (GUI) connected to Matlab (Mathworks Inc., USA) *via* a Universal Serial Bus (USB).

The portable readout system employed time-multiplexed signal acquisitions (MUX) from the 12 channels connected to each graphene sensing unit, whereby the individual drain-source currents (iDS) were detected in a voltage-divider circuit topology, amplified, and digitized by a high-speed and high-resolution analogue-to-digital converter (ADC) connected to the central microcontroller (MCU) on the embedded electronics. The drain-source voltage level (vDS) on the graphene channels is imposed by the user during the iDS measurements through the GUI, which converts the digital value into an analogue equivalent level using digital-to-analogue conversion (DAC) circuitry within the range ± 8V and resolution of 250 µV. For measurement of the iDS – vGS curve, also known as Dirac's point, an additional DAC is employed to provide an automatic voltage level sweep across the floating gate and within the range specified by the user. iDS measurements in both stimulation modes (including extended temporal measurements) can be applied to a single graphene channel or to all twelve channels at once, with the true value for iDS being calculated by a Fast Fourier Transform (FFT) algorithm applied to the samples acquired by the ADC, thereby achieving a current resolution of 20 nA and bandwidth of 15 kHz.

S 2 Functionalization and characterisation of graphene surface

Various characterization techniques were performed to examine the functionalization of TCPP and antibody on the graphene surface.

The Raman spectra (Figure S7) recorded for bare graphene showed peaks at 1580 cm^{-1}, which is commonly attributed to the G band of sp^2 carbon in graphene. In addition, the presence of peaks at 1350 cm^{-1} (D band), 2700 cm^{-1} (2D secondary D band) is also shown, indicating a high quality of monolayer graphene. Raman spectroscopy analysis of the graphene surface after functionalization with TCPP showed the characteristic peaks of TCPP,[1] which are the vibrations of C-N (1240 cm^{-1}), C-H (1454 cm^{-1}), C-C (1565 cm^{-1}), and a weak shoulder peak (1496 cm^{-1}). The Raman D-to-G peak of the graphene increased from 0.32 (bare graphene) to 0.89 (after functionalization with TCPP), indicating that there is some disorder in the graphene sheet. The intensity ratio I_{2D}/I_G = 1.89 indicates high-quality graphene after the functionalization process.

X-ray photoelectron spectroscopy (XPS) was used to confirm the existence of TCPP and antibody on the graphene surface. GPC-1 antibodies were selected as a pancreatic cancer exosome biomarker targeting GPC-1 proteins on the surface of cancer exosomes.

Figure S7.shows the changes in principle C 1s and N 1s core levels after each functionalization step. The high-resolution N 1s spectra show a significant increase in the N 1s peak at 399.8 eV

after GPC-1 antibody conjugation. The high-resolution C 1s spectrum is composed of 4 components at the surface, C–C at 284.9 eV, C–O at 285.9 eV, C–N at 286.4 eV, and O–C═O at 287.7 eV, corresponding to the large number of amine and amide bonds present in the antibodies.

Atomic force microscopy was used to reveal changes in the surface morphology and quality of graphene after each step of the functionalization process (Figure S7). The surface roughness of pristine graphene is measured to be 0.3 nm. After surface treatment with 2 h of TCPP, the surface roughness increased to 0.41 nm. After antibody modification, the surface roughness further increased to 0.68 nm.

These results suggest that the graphene surface was successfully functionalized and can be used for sensing target exosomes.

S 3 Selection of antibody for PDAC cancer exosome detection

Exosomes are extracellular vesicles that carry proteins, nucleic acids, and lipids. Many exosome-enriched proteins have been reported. Specific exosomal enriched proteins are expressed more on pancreatic cancer cell-derived exosomes compared to healthy ones, which offers the possibility to diagnose and distinguish patients with PC.[2 - 4] Protein biomarkers enriched on pancreatic cancer-cell-derived-exosomes include glypican-1 (GPC-1),[2] RHOB,[5] CD63 and Prom1.[6] All of these proteins can be expressed on both cancerous and healthy exosomes.[4] In order to choose an appropriate biomarker for detection using GFETs, the protein should be highly expressed on cancerous exosomes with low expression on healthy exosomes. Therefore, the selectivity of each protein biomarker to cancer cell-derived compared to healthy exosomes was tested. Four types of antibodies (CD63, GPC-1, Prom1, and RhoB) were immobilized on the graphene surface to investigate their sensitivity and selectivity to PDAC cancer exosomes in patient plasma samples (see section S2 for details of the functionalization protocol for the graphene surface). Figure S8 summarizes the performance of these antibodies in differentiating PDAC cancer exosomes from healthy exosomes in blood plasma. Using AFM, it was found that all protein biomarkers are abundant on the PDAC cancerous exosomes. All the selected protein biomarkers were found to have increased expression in plasma exosomes in PDAC patients compared to the healthy controls. Furthermore, CD63 antibodies exhibited the highest capture rate for both cancerous and healthy exosomes. These results were expected, because CD63 has been observed to be enriched on all exosomal membranes.[7,8] The high concentration of CD63+ exosomes confirmed the presence of exosomes in the tested samples.[7,9,10] We found that Prom1 antibodies have low selectivity, while that of RHOB was higher. However, we also found that the GPC-1 antibodies had the highest selectivity to the PDAC cancer exosomes and the lowest capture number of healthy exosomes (Figure S8), showing significantly higher specificity to the PDAC cancer exosomes. The specificity of each protein for cancer and healthy exosomes were 1.4:1, 3.2:1, 1.6:1, and 1.8:1 for CD63, GPC-1, Prom1 and ROHB respectively.

S 4 Dynamic light scattering (DLS)

Dynamic light-scattering measurements were performed using a Zetasizer Nano ZS (Malvern Instruments Ltd, Worcestershire, UK). The intensity, volume, and distribution data for each sample were collected on a continuous basis for 4 minutes in sets of three. Three different measurements from the isolated exosome samples were performed.

Supplementary Figures

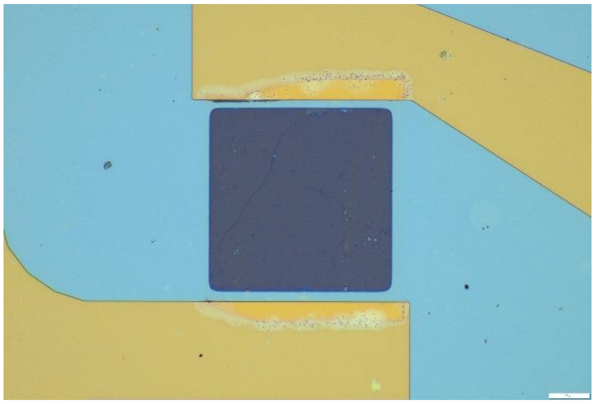

Figure S1. Illustration of the optical image of on-chip integrated GFET devices.

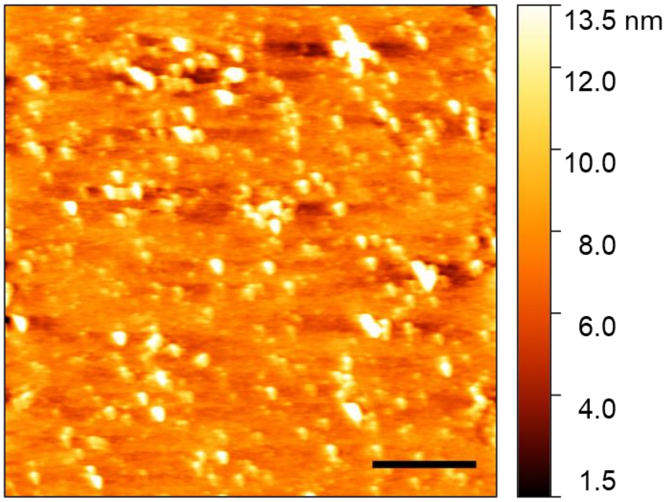

Figure S2. Atomic force microscopy images of GPC-1 Ab functionalized graphene surface incubated with MCF-7 exosomes (model exosomes) in buffer, suggesting the expression of GPC-1 on the surface of MCF-7 exosomes.

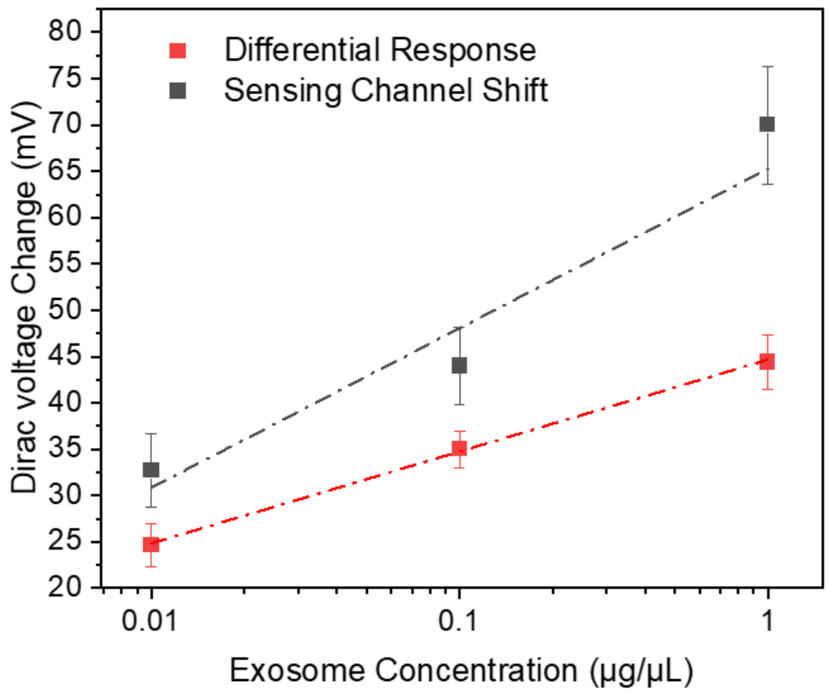

Figure S3. Calibration curve of the GFET biosensor for detection of various concentrations of spiked model exosomes in plasma. Error bars are determined by the standard deviation of multiple device measurements, $4 \leq n \leq 24$ for each reading. Differential response represents the device signal, which is the difference between sensing channel and control channel.

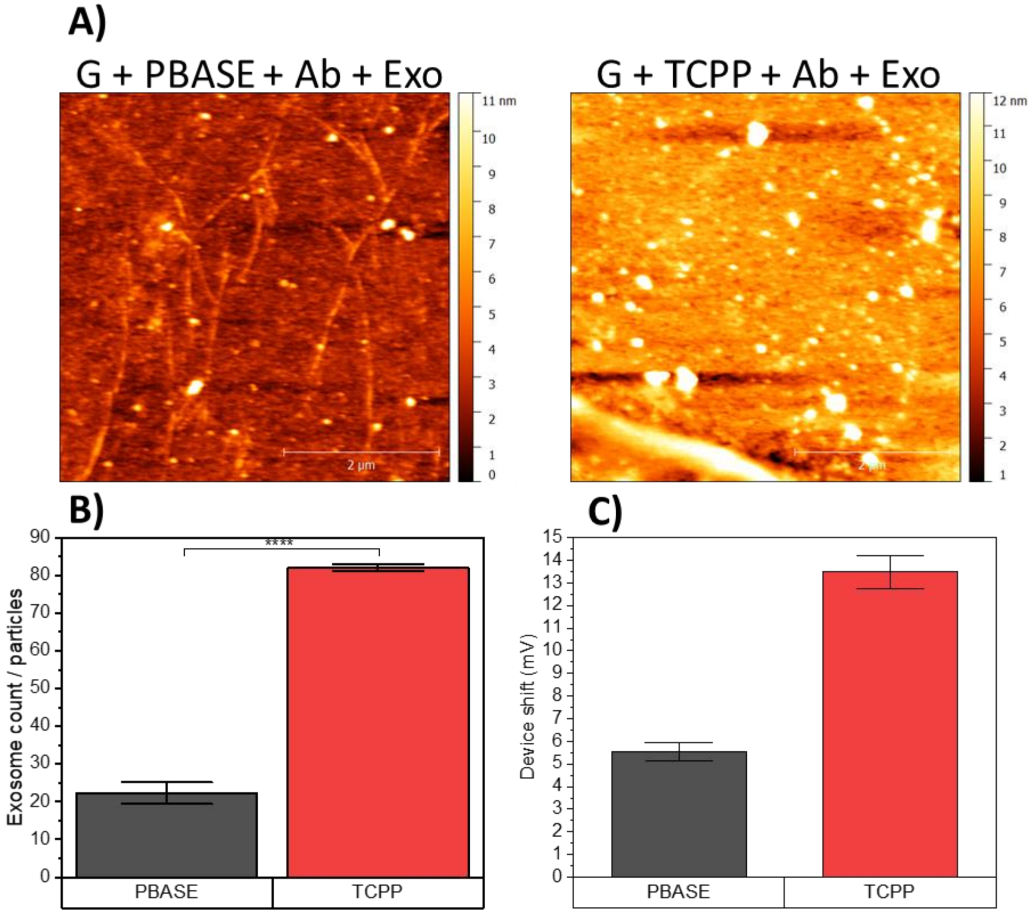

Figure S4. AFM profile of exosome expression on PBASE and TCPP decorated graphene substrate with CD63 antibodies, after 20 min incubation with model exosomes in buffer. (0.1 µg/ µL) AFM scale bars = 1 µm. B) Numbers of particles captured by antibodies functionalized on 25 µm2 of graphene surface after 20 min incubation with model exosomes in buffer (****P < 0.0001, n = 4). Data are mean ± s.d. C) Comparison of sensing performance of GFET devices functionalized with two different linker molecules, PBASE and TCPP. Directly after surface functionalization, the surface of GFET devices is immobilized with CD63 antibodies. Transfer characteristic measurements were performed on GFET sensors for detection of spiked model exosomes in buffer. (1 ng/ µL) Error bars are determined by the standard deviation of multiple device measurements, $3 \leq n \leq 6$ for each reading.

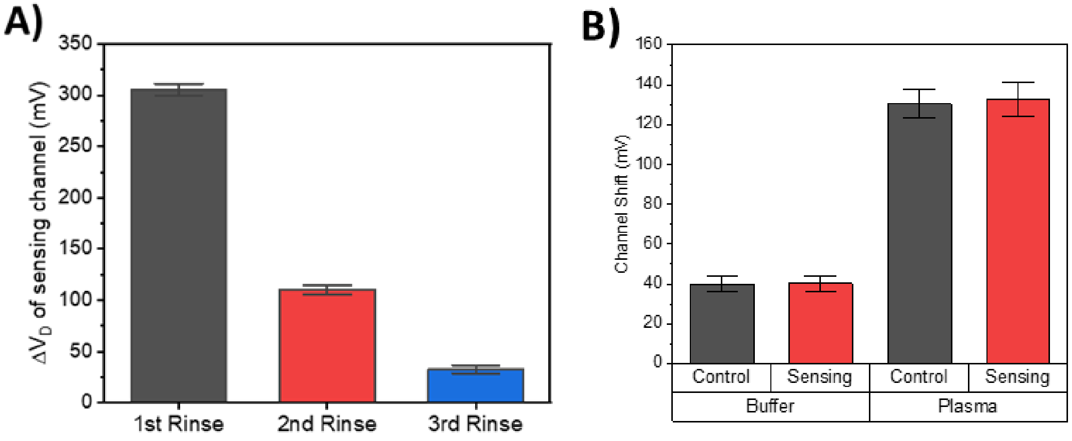

Figure S5. A) Comparison of device shift from sensing channel with different times of rinse. The decrease in device shift is due to the complex environment of blood plasma samples. The high salinity and sticky nature of blood plasma led to salt accumulation and left unwanted residues on the surface of GFET sensors B) Comparison of channel shift in control and sensing channels when testing with pure buffer and healthy plasma sample.

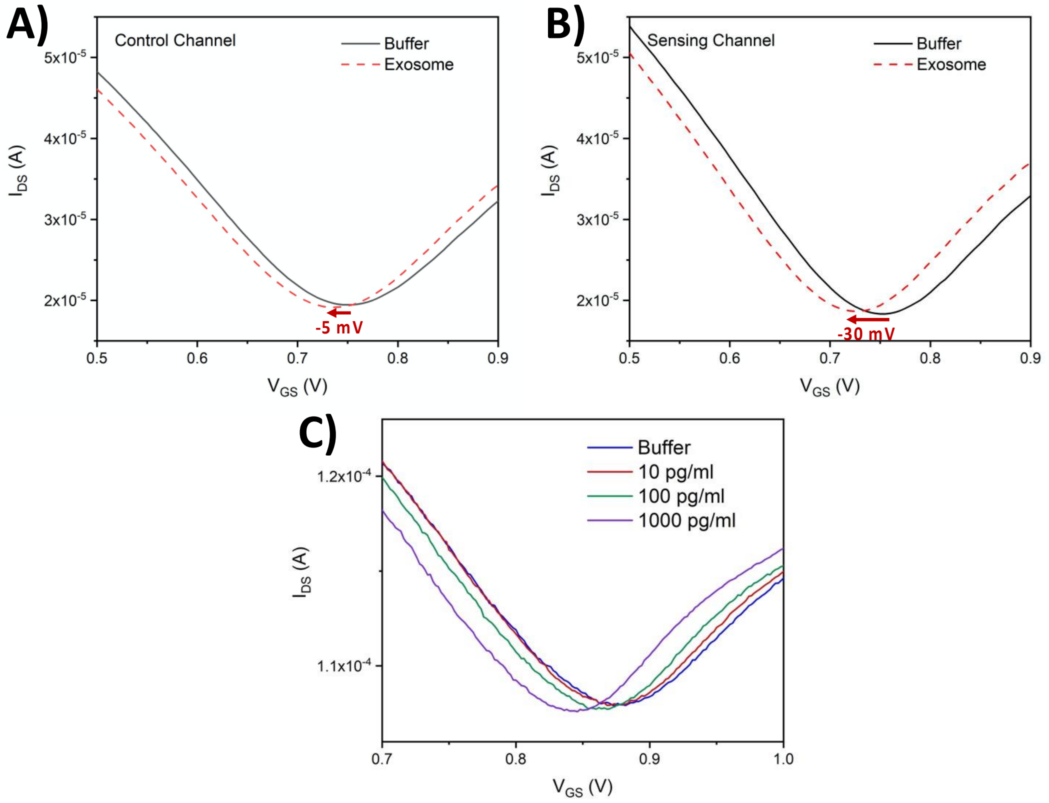

Figure S6. Validation of GFET biosensor for detection of exosome with protocol of using control and sensing channel. Detection of cancer exosomes in buffer using the GFET biosensor. Representative I_{DS}-V_{GS} curve of the A) control channel and B) sensing channel on the GFET biosensor after exosome binding. Significant curve shift seen in the sensing channel in comparison to the control channel, suggesting that the shift arise from exosome binding on sensing channel. C) Representative I_{DS}-V_{GS} curve of the sensing channel of GFET biosensor in buffer with various concentrations of exosomes.

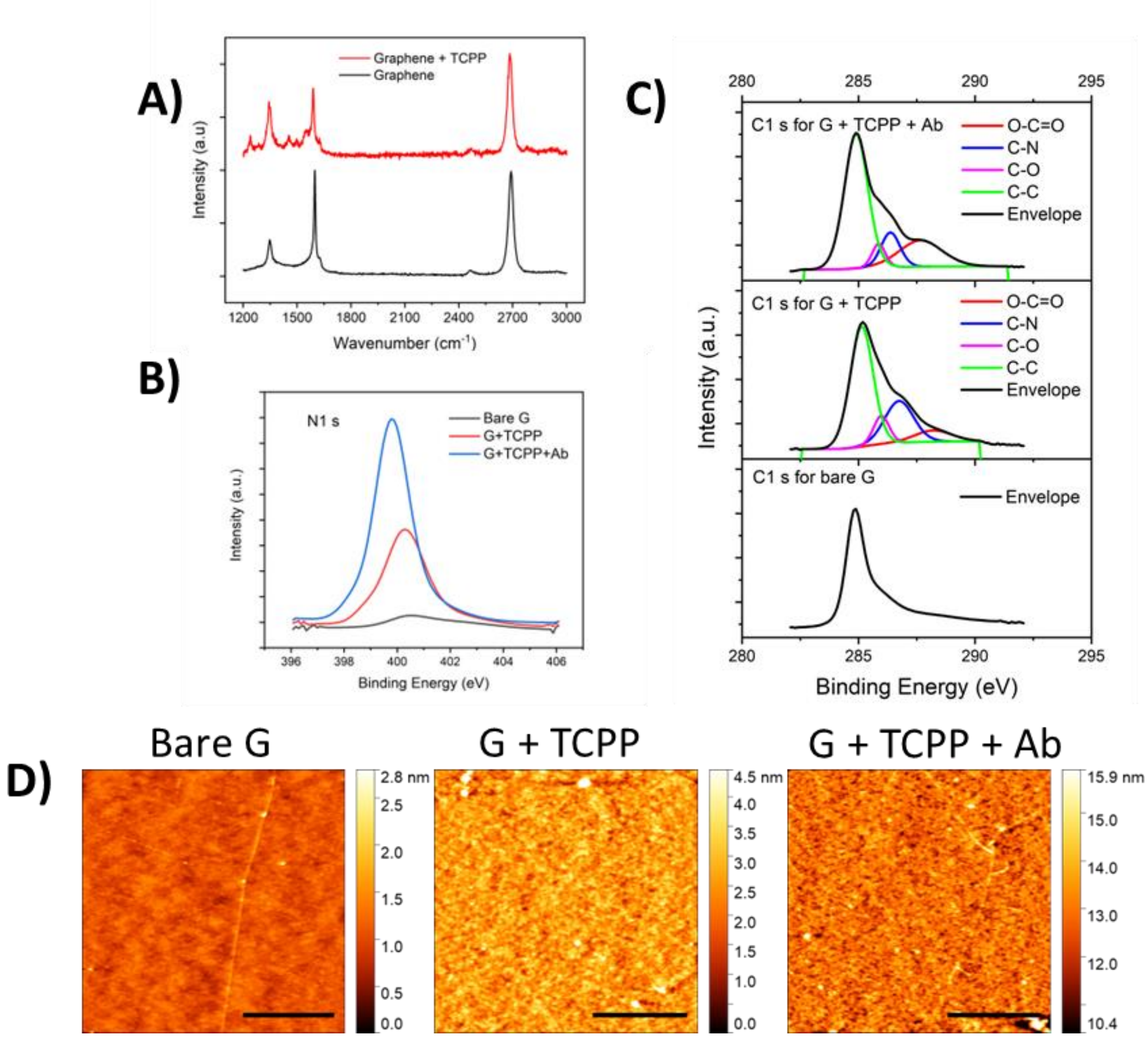

Figure S7. Characterization of surface functionalization of the GFET biosensor: A) Raman spectra of bare graphene and TCPP-functionalized graphene. B) N1 s and C) C1 s X-ray photoelectron spectroscopy (XPS) spectra of graphene with different degrees of functionalization: bare, with TCPP, and with TCPP + antibody respectively. D) Atomic force microscopy (AFM) profile for characterization of bare graphene, TCPP modified graphene, and graphene surface after TCPP + antibody biofunctionalization. AFM scale bars = 1 μm.

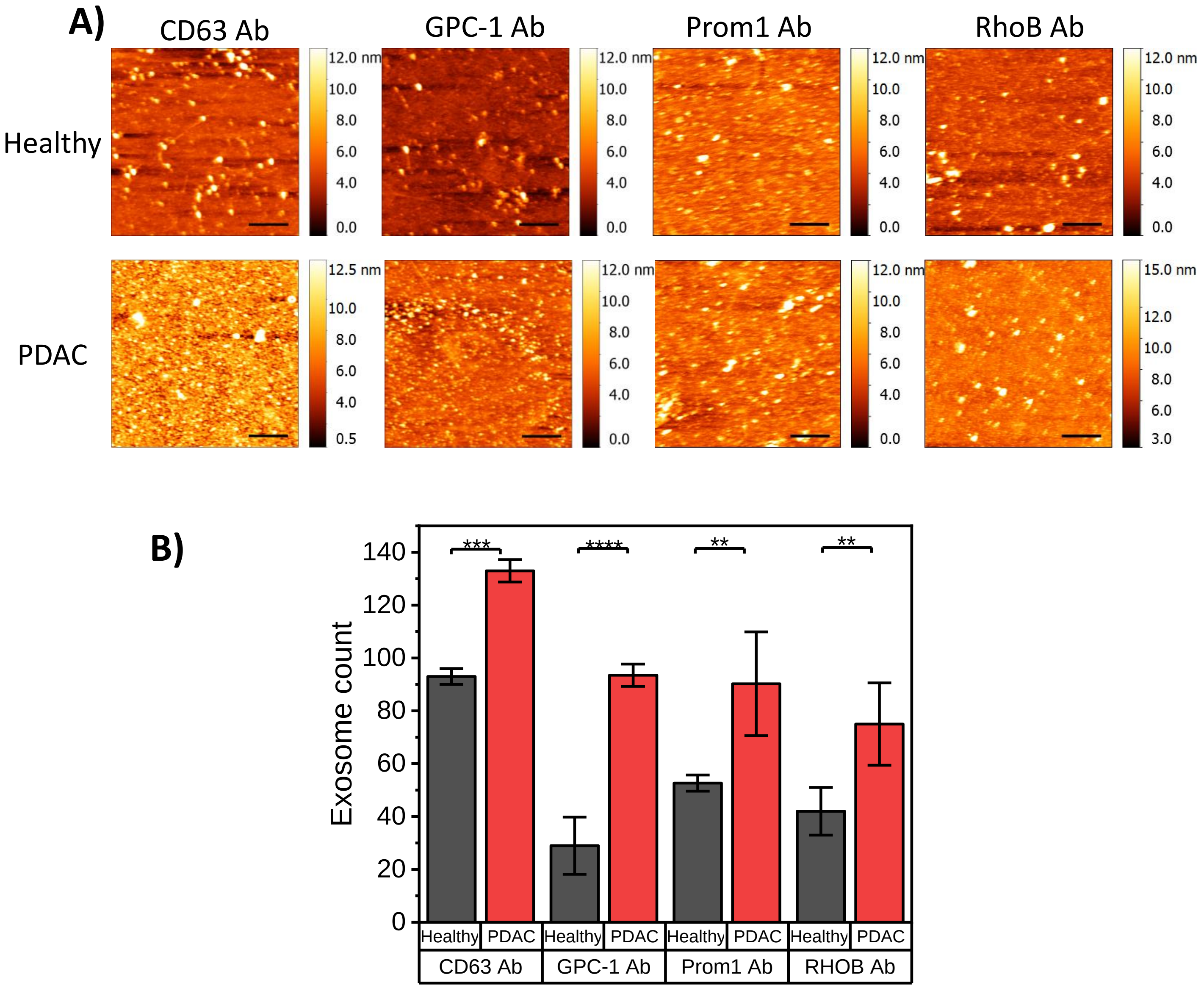

Figure S8. Selection and validation of antibodies for the clinical detection of pancreatic cancer cell-derived exosomes in patient plasma: A) AFM profile of exosome expression on 4 different antibodies functionalized on TCPP decorated graphene substrate (CD63, GPC-1, Prom1, and RhoB antibodies) after 20 min incubation with healthy plasma and PDAC patient plasma. AFM scale bars = 1 μm. B) Numbers of particles captured by CD63, GPC-1, Prom1, and RhoB antibodies functionalized on 25 μm^2 of graphene surface after 20 min incubation with healthy plasma and PDAC patient plasma (**$P < 0.01$, ***$P < 0.001$, ****$P < 0.0001$, $n = 4$). Data are mean ± s.d.

Supplementary Table

Table S1. Summary of the patient cohort.

	PDAC group	Healthy control group
**Number of patients**	18	8
**Age-yrs.**		
**Median**	69	69.5
**Range**	49 - 84	60 - 74.5
**BMI (kg/m^2)**	24.64	n/a
**Gender**		
**Male**	10 (55.6%)	4 (50%)
**Female**	8 (44.4%)	4 (50%)
**Smoking**	8 (44.4%)	2 (25%)
**Tumour size – mm**		
**Median**	27.5	n/a
**Range**	15 - 38	n/a
**Diagnosis**		
**PDAC Stage**	**no. (%)**	**Benign**
**I**	1 (5.56%)	gallstones, n=3
**II**	1 (5.56%)	gallstone pancreatitis, n=1
**III**	7 (38.9%)	duodenal diverticulum, n=1
**IV**	7 (38.9%)	duodenal mucosal cyst, n=1
**V**	1 (5.56%)	Papilla fibrosis, n=1
**Metastatic**	1 (5.56%)	fatty infiltration of pancreas, n=1

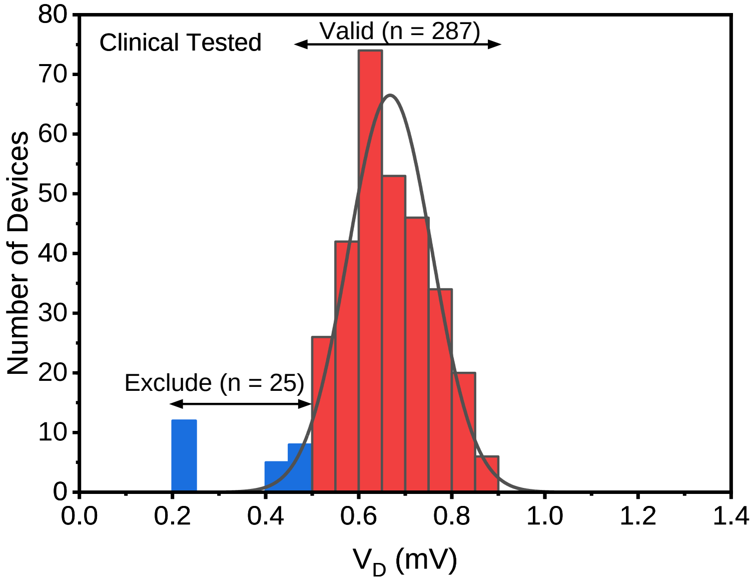

Figure S9. Device exclusion criteria for GFET reproducibility and validity. Results from n = 281 clinically tested devices that satisfied the exclusion criteria are included in the study. Devices (n=25) with $V_D < 0.47\,V$ or $V_D > 0.95\,V$ were excluded in this study. Devices (n=12) that exhibited values of $R\ > 1700\ \Omega\ or\ R < 700\ \Omega$ were also excluded.

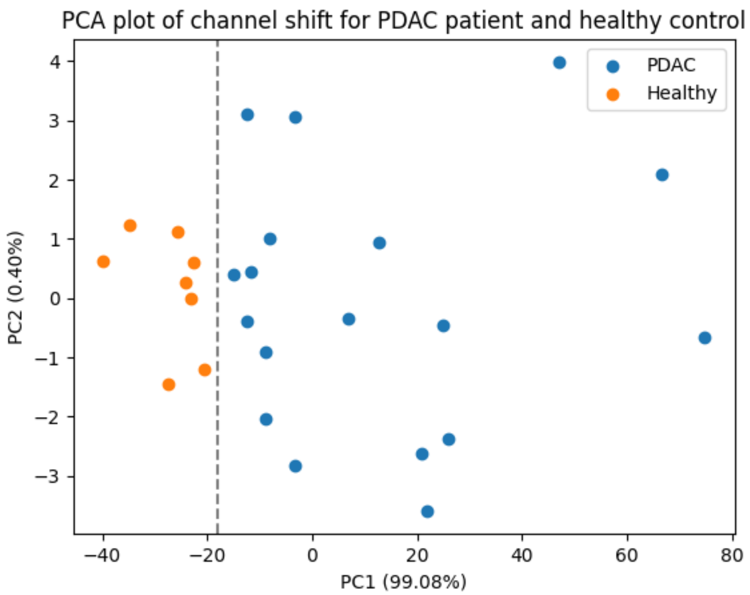

Figure S10. Principal component analysis (PCA) plot of the relevant channel shift signal for PDAC and control group. PC1 captured the greatest variance of the data set, (99.08%). A clear separation line can be drawn on the PC1 of the PCA plot. This separation line can well separate the healthy control from the PDAC patient.

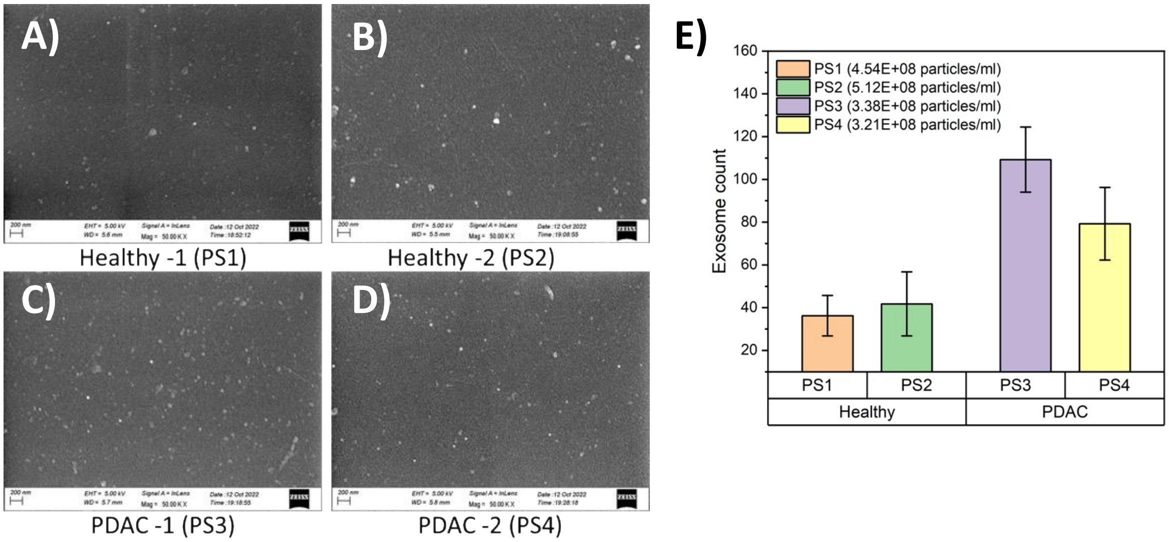

Figure S11. Comparison of exosomes captured on functionalized GFET sensors, scanning electron microscopy analysis of exosomes from healthy plasma samples: A) PS1 and B) PS2 and PDAC plasma samples. C) PS3 and D) PS4 captured on the functionalized graphene surface. SEM scale bars = 200 nm. E) Exosome counts on graphene incubated with different samples, taken from an average of four spots on each graphene surface.

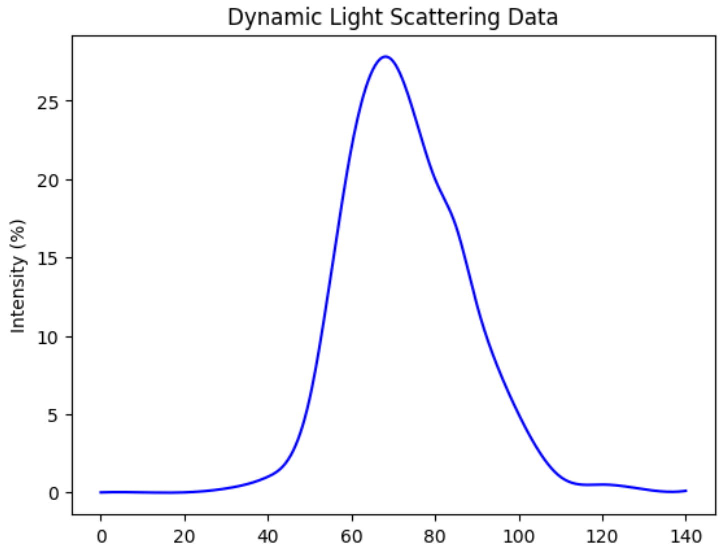

Figure S12. Dynamic light scattering measurements of isolated exosomes from blood plasma samples. The DLS showed an average size of 76.5 nm, with the size of particles lies between 30 and 130 nm.

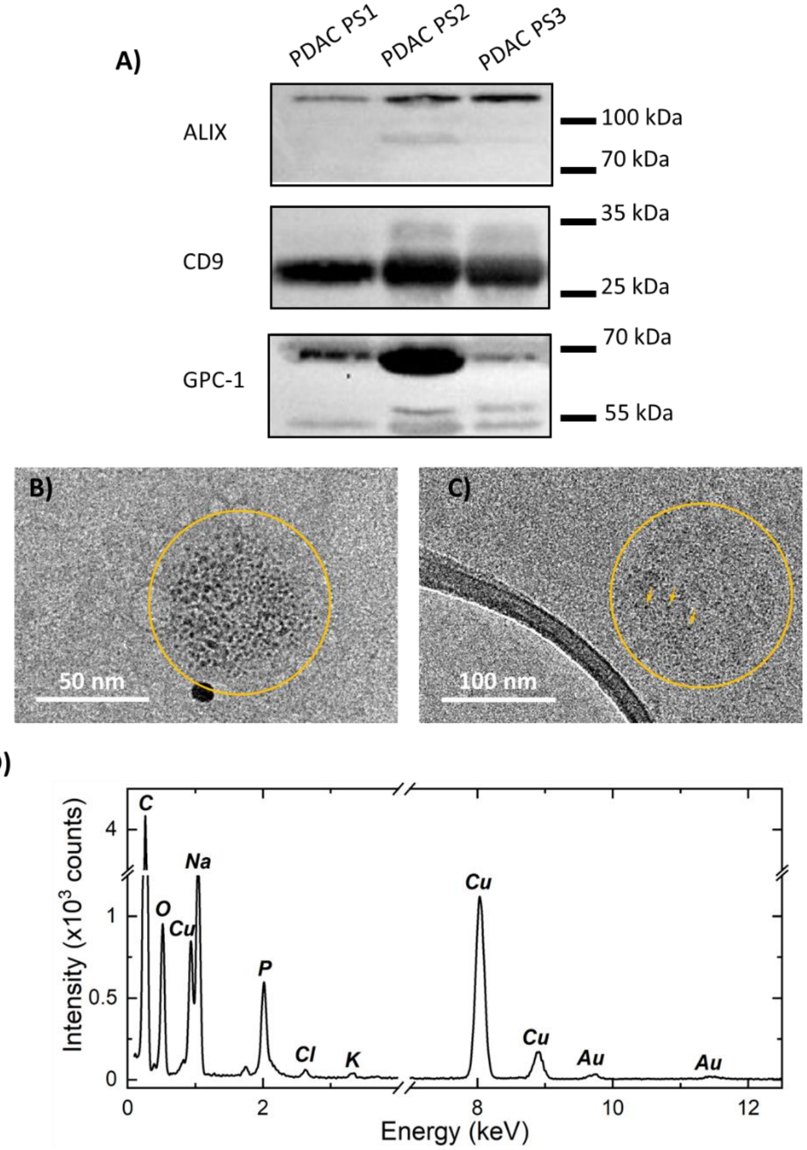

Figure S13. A) Western blot analyses of exosomes isolated from 3 PDAC patients' plasma, with ALIX antibody, CD9 antibody, and GPC-1 antibody. B) Low-magnification IG-TEM image of GPC-1 in PDAC cancer exosomes with immunogold labelling. The outlines of individual exosomes are highlighted by dashed yellow line for clarity. C) Low-magnification IG-TEM image of a healthy exosome with immunogold labelling. The yellow circles around individual exosomes are given for visual guidance. The arrows point to the individual Au particles on the surface of a healthy exosome. D) EDX spectrum of an Au-stained P-exosome showing the Au signal. The Cu signal originates from the TEM grid.

References

(1) Cotton, T. M.; Schultz, S. G.; Van Duyne, R. P. Surface-Enhanced Resonance Raman Scattering from Water-Soluble Porphyrins Adsorbed on a Silver Electrode. *Journal of the American Chemical Society* **1982**, *104* (24), 6528–6532. https://doi.org/10.1021/ja00388a008.

(2) V. S.; Mittendorf, E. A.; Weitz, J.; Rahbari, N.; Reissfelder, C.; Pilarsky, C.; Fraga, M. F.; Piwnica-Worms, D.; Kalluri, R. Glypican-1 Identifies Cancer Exosomes and Detects Early Pancreatic Cancer. *Nature* **2015**, *523* (7559), 177–182. https://doi.org/10.1038/nature14581.

(3) Hu, C.; Jiang, W.; Lv, M.; Fan, S.; Lu, Y.; Wu, Q.; Pi, J. Potentiality of Exosomal Proteins as Novel Cancer Biomarkers for Liquid Biopsy. *Front. Immunol.* **2022**, *13*. https://doi.org/10.3389/fimmu.2022.792046.

(4) Wang, X.; Huang, J.; Chen, W.; Li, G.; Li, Z.; Lei, J. The Updated Role of Exosomal Proteins in the Diagnosis, Prognosis, and Treatment of Cancer. *Exp. Mol. Med.* **2022**, *54* (9), 1390–1400. https://doi.org/10.1038/s12276-022-00855-4.

(5) Servage, K. A.; Stefanius, K.; Gray, H. F.; Orth, K. Proteomic Profiling of Small Extracellular Vesicles Secreted by Human Pancreatic Cancer Cells Implicated in Cellular Transformation. *Sci. Rep.* **2020**, *10* (1). https://doi.org/10.1038/s41598-020-64718-6.

(6) Ruivo, C. F.; Bastos, N.; Adem, B.; Batista, I.; Duraes, C.; Melo, C. A.; Castaldo, S. A.; Campos-Laborie, F.; Moutinho-Ribeiro, P.; Morão, B.; Costa-Pinto, A.; Silva, S.; Osorio, H.; Ciordia, S.; Costa, J. L.; Goodrich, D.; Cavadas, B.; Pereira, L.; Kouzarides, T.; Macedo, G.; Maio, R.; Carneiro, F.; Cravo, M.; Kalluri, R.; Machado, J. C.; Melo, S. A. Extracellular Vesicles from Pancreatic Cancer Stem Cells Lead an Intratumor Communication Network (EVNet) to Fuel Tumour Progression. *Gut* **2022**, gutjnl-2021. https://doi.org/10.1136/gutjnl-2021-324994.

(7) Lötvall, J.; Hill, A. F.; Hochberg, F.; Buzás, E. I.; Di Vizio, D.; Gardiner, C.; Gho, Y. S.; Kurochkin, I. V.; Mathivanan, S.; Quesenberry, P.; Sahoo, S.; Tahara, H.; Wauben, M. H.; Witwer, K. W.; Théry, C. Minimal Experimental Requirements for Definition of Extracellular Vesicles and Their Functions: A Position Statement from the International Society for Extracellular Vesicles. *J. Extracell. Vesicles* **2014**, *3* (1), 26913. https://doi.org/10.3402/jev.v3.26913.

(8) Andreu, Z.; YÃ¡Ã±ez-MÃ³, M. Tetraspanins in Extracellular Vesicle Formation and Function. *Front. Immunol.* **2014**, *5*. https://doi.org/10.3389/fimmu.2014.00442.

(9) Liu, C.; Xu, X.; Li, B.; Situ, B.; Pan, W.; Hu, Y.; An, T.; Yao, S.; Zheng, L. Single-Exosome-Counting Immunoassays for Cancer Diagnostics. *Nano Lett.* **2018**, *18* (7), 4226–4232. https://doi.org/10.1021/acs.nanolett.8b01184.

(10) Ramadan, S.; Lobo, R.; Zhang, Y.; Xu, L.; Shaforost, O.; Kwong Hong Tsang, D.; Feng, J.; Yin, T.; Qiao, M.; Rajeshirke, A.; Jiao, L. R.; Petrov, P. K.; Dunlop, I. E.; Titirici, M.-M.; Klein, N. Carbon-Dot-Enhanced Graphene Field-Effect Transistors for

Ultrasensitive Detection of Exosomes. *ACS Appl. Mater. Interfaces* **2021**, *13* (7), 7854–7864. https://doi.org/10.1021/acsami.0c18293.
